# Motility and chemotaxis of bacteria-driven microswimmers fabricated using antigen 43-mediated biotin display

**DOI:** 10.1038/s41598-018-28102-9

**Published:** 2018-06-28

**Authors:** Oliver Schauer, Babak Mostaghaci, Remy Colin, Daniel Hürtgen, David Kraus, Metin Sitti, Victor Sourjik

**Affiliations:** 1 Department of Systems and Synthetic Microbiology, Max Planck Institute for Terrestrial Microbiology & LOEWE Center for Synthetic Microbiology (SYNMIKRO), 35043 Marburg, Germany; 2 Physical Intelligence Department, Max Planck Institute for Intelligent Systems, 70569 Stuttgart, Germany

## Abstract

Bacteria-driven biohybrid microswimmers (bacteriabots) combine synthetic cargo with motile living bacteria that enable propulsion and steering. Although fabrication and potential use of such bacteriabots have attracted much attention, existing methods of fabrication require an extensive sample preparation that can drastically decrease the viability and motility of bacteria. Moreover, chemotactic behavior of bacteriabots in a liquid medium with chemical gradients has remained largely unclear. To overcome these shortcomings, we designed *Escherichia coli* to autonomously display biotin on its cell surface via the engineered autotransporter antigen 43 and thus to bind streptavidin-coated cargo. We show that the cargo attachment to these bacteria is greatly enhanced by motility and occurs predominantly at the cell poles, which is greatly beneficial for the fabrication of motile bacteriabots. We further performed a systemic study to understand and optimize the ability of these bacteriabots to follow chemical gradients. We demonstrate that the chemotaxis of bacteriabots is primarily limited by the cargo-dependent reduction of swimming speed and show that the fabrication of bacteriabots using elongated *E. coli* cells can be used to overcome this limitation.

## Introduction

Cell-driven biohybrids have recently gained much attention due to their potential biomedical applications, including the targeted active delivery of cargo, such as drug, gene, or imaging contrast agent^1–9^. Here, unicellular organisms, such as bacteria or algae, or cells of higher eukaryotes (*e.g*., cardiomyocytes or spermatozoa), are used to propel the biohybrid swimmers in stagnant or low-velocity physiological fluids. These microswimmers can also sense and follow environmental gradients of chemicals, light, pH, magnetic fields, or oxygen^1,10,11^. Bacteria-driven microswimmers (bacteriabots) are especially promising due to the diversity of their sensory and tactic behaviors, high and robust motility in liquid media at diverse environmental conditions (*i.e*., variable temperature, pH, oxygen concentration etc.) and therapeutic^12^ and targeting^13^ capabilities for specific diseases^14^, as well as the comparative ease of their genetic modification. Recent studies have shown that bacteriabots based on *Escherichia coli, Serratia marcescens* or *Salmonella enterica* serovar Typhimurium can in principle follow chemoattractant gradients^15–19^, including gradients toward cancerous cells^3,20^. However, reliable application of such bacteriabots, in biomedicine and beyond, requires better fundamental understanding of their chemotactic behavior, considering that attachment of single or multiple bacteria to synthetic bodies, such as microparticles^6,14,16–18,21^, microsheets^22^, microemulsions^7^, and microtubes^23^, is likely to affect their taxis performance compared to the free swimming bacteria.

The best-studied model organism for bacterial chemotaxis is *E. coli*^24^. Similar to other chemotactic bacteria, swimming *E. coli* cells make temporal comparisons of their environment and modulate frequency of changes in the swimming direction dependent on whether the environment becomes more or less favorable^25–27^. This is controlled by the direction of their flagellar motor rotation, with counterclockwise rotation resulting in more-or-less straight runs powered by a bundle of multiple flagella, and clockwise rotation leading to a partial disintegration of the bundle and cell tumbling. The underlying chemotaxis signaling network of *E. coli* consists of five receptors (Tar, Tsr, Tap, Trg, and Aer), which are arranged in chemoreceptor clusters together with two cytoplasmic proteins, the adaptor CheW and the kinase CheA^28,29^. Further cytoplasmic signaling proteins are the response regulator CheY, its phosphatase CheZ, and the receptor methylation/demethylation enzymes CheR and CheB. The main function of the sensory clusters is to process environmental stimuli and to provide a coordinated output – the level of CheY phosphorylation – controlling the direction of flagellar motor rotation. The wide dynamic range of stimulus discriminations in *E. coli* chemotaxis is maintained by the activity-dependent methylation of chemotaxis receptors. Receptor methylation compensates stimulus-induced changes in activity of the receptor-associated kinase CheA, so that bacteria can adapt to constant background stimulation. Additionally, due to its delayed occurrence, receptor methylation also serves as a short-term memory, enabling the aforementioned temporal comparisons of environmental conditions. The interplay between the function of the chemotaxis system and flagellar motility has been apparently fine-tuned by evolution to enable optimal chemotaxis^24,30,31^. In contrast to this excellent understanding of chemotaxis and motility of free bacterial cells, it remains largely unclear how bacterial chemotaxis strategy is affected in bacteriabots by attachment of synthetic cargo.

Similarly, critical for efficient fabrication and application of bacteriabots is attachment of the bacterial cell to the synthetic cargo (*e.g*., microparticle). Although electrostatic or hydrophobic interactions provide the simplest ways to attach bacterial cells to particles^6,21,32^, such attachment may not be specific and reliable enough for biomedical applications where bacteriabots need to maintain their integrity in biological fluids with high concentrations of proteins, ions and possible fluidic shear forces^12^. In contrast, the biotin-streptavidin interaction is highly specific and very strong^33^. However, although it has been utilized in the fabrication of bacteriabots^7,15,34^ by chemical functionalization of the bacterial cell surface with biotin^20,35^ or by using a biotin-conjugated antibody directed against surface lipopolysaccharides^36^, the yield and efficiency of motile bacteriabots fabricated using these methods remained low, because several functionalization and washing steps negatively affect bacterial motility and viability. Moreover, the specificity of these methods is rather limited^37^.

To address these shortcomings in the fabrication and application of bacteriabots, here we first developed a fast, specific, and efficient labeling approach of the microparticle attachment. This assay relies on genetically modified autotransporter antigen 43 (Ag43)^38^ to display biotin on *E. coli* surface and utilizes bacterial motility to greatly accelerate the attachment of streptavidin-coated cargo particles. We subsequently characterized bacteriabot chemotaxis, showing that it is primarily limited by the decrease in the swimming speed upon microparticle attachment. Finally, we show that this limitation could be largely circumvented by controlled elongation of *E. coli* cell body, which enables both faster particle propulsion and much more efficient chemotaxis, thus holding high potential for future biomedical applications.

## Results

### Surface display of biotinylated peptide by autotransporter Ag43

**Figure 1:**
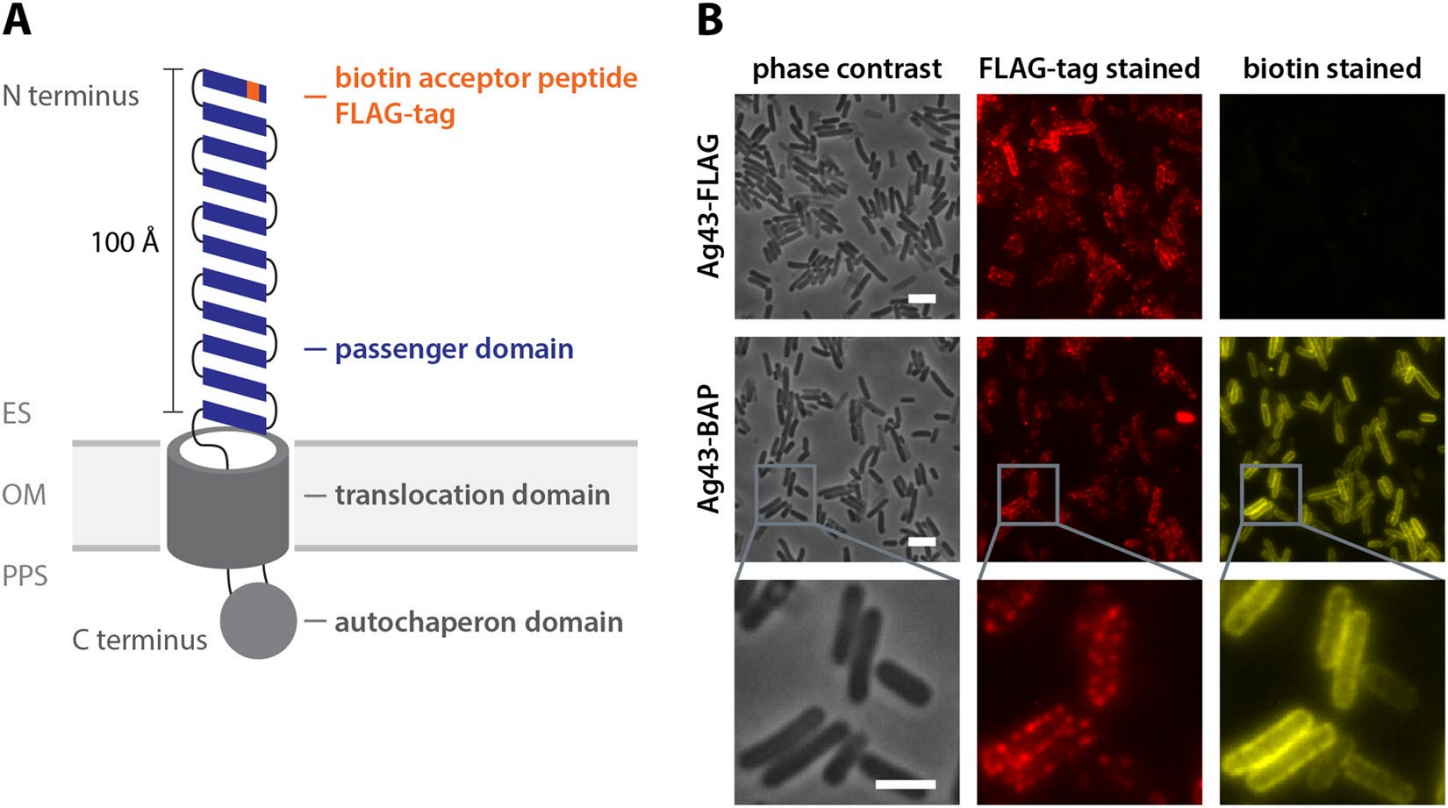
Biotin display on the cell surface of *E. coli* via recombinant Ag43. (**A**) Schematic model of the Ag43-mediated peptide display. The N-terminus of Ag43 was modified with a FLAG epitope tag and a biotin acceptor peptide (BAP). ES: extracellular space, OM: outer membrane, PPS: periplasmic space. (**B**) *E. coli* cells carrying the recombinant Ag43-FLAG (induced with 100 µM isopropyl-β-D-thiogalactopyranoside (IPTG)) with and without BAP were analyzed using anti-FLAG immunostaining (red) and NeutrAvidin-biotin staining (yellow) for detecting recombinant Ag43-FLAG and surface-displayed biotin, respectively, and subsequent fluorescence microscopy. Scale bar: 4 µm.

Attaching bacteria to abiotic surfaces is the first challenge in the bacteriabot construction. To enable specific attachment of *E. coli* to a streptavidin-coated surface, we utilized the autotransporter Ag43 for the cell-surface display of biotin. Ag43 is involved in cell-cell autoaggregation of *E. coli*, and with about 50,000 copies per cell, it is one of the most abundant outer membrane proteins^39^. The N-terminal part of the protein can hold modified peptides and protrudes about 100 Å from the outer membrane, beyond the ~20 Å lipopolysaccharide layer, thus making it accessible to extracellular molecules^40–44^. We genetically modified the N-terminus of Ag43 with a biotin acceptor peptide (BAP), which can be biotinylated intracellularly by the native biotin ligase BirA^45,46^. After successful translocation through the inner cell membrane and insertion into the outer membrane, the biotinylated N-terminus of Ag43 should be displayed on the cell surface (Fig. 1A). As an additional control of protein translocation, our construct also contained a FLAG epitope.

Consistent with our expectation, the passenger domain of the recombinant Ag43 (Ag43-BAP) could be detected on the surface of most cells, visible as the FLAG-tag antibody staining of the outer membrane (Fig. 1B). Same cells also showed pronounced biotin staining, indicating that BAP was biotinylated in the cytoplasm and biotin was accessible to fluorescently labeled NeutrAvidin (analogue of streptavidin). The construct showed polar localization in up to 35% of the cells, whereas the majority of cells showed rather uniform distribution of the label (Supplementary Fig. S1). Biotinylation of Ag43-BAP could also be quantified using flow cytometry (Supplementary Fig. S2). It was apparently partly limited by the availability of the endogenous biotin, since addition of biotin to the growth medium increased staining by ~4 fold. The incubation of cells with the exogenously added BirA and biotin after cell harvest led to a further increase in the biotinylation of Ag43-BAP, suggesting that even in presence of biotin in the growth medium, *E. coli* biotinylates only a fraction of Ag43-BAP. Nevertheless, as the natural biotinylation was sufficient for efficient attachment of microparticles (see below), it was used in all further experiments.

### Microparticle attachment and bacterial motility

**Figure 2:**
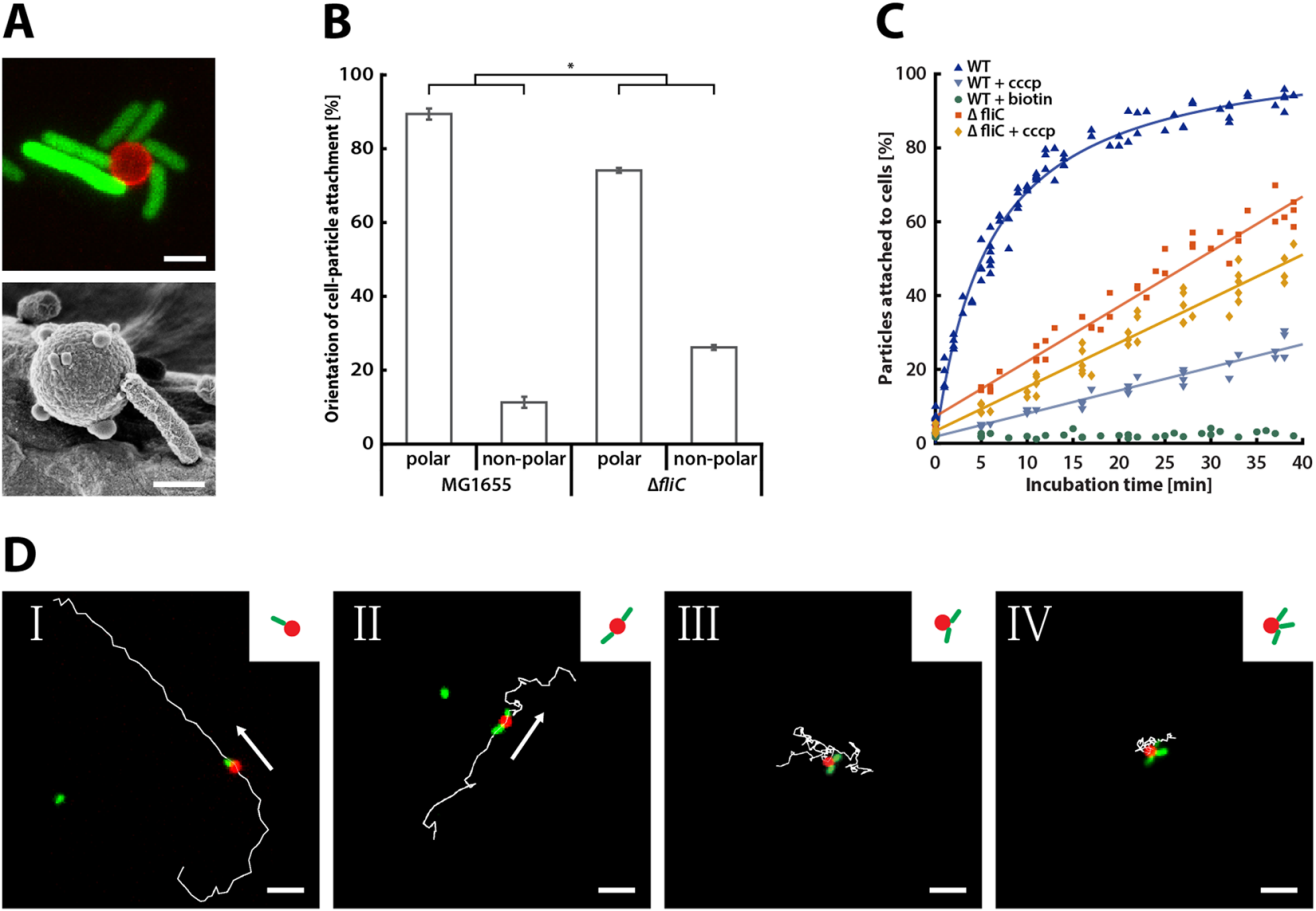
Microparticle attachment and motility of the fabricated bacteriabots. Motile wild-type *E. coli* cells (WT) and non-motile Δ*fliC* cells carrying the recombinant Ag43-BAP and an inducible GFP construct were incubated for 20 min with streptavidin-coated 2.2-µm PMMA particles, at a mixing ratio of 1:30. (**A**) Images of particle-attached cells acquired via confocal laser scanning microscopy (top) or scanning electron microscopy (bottom), with scale bars being 2 µm and 1 µm, respectively. For better visualization, cells were elongated by inhibiting cell division using cephalexin to the growth medium for one hour before harvesting. (**B**) Corresponding quantification of the polar and non-polar cell-particle attachment of WT (for 786 attached cells) and Δ*fliC* (for 1115 attached cells) cells were analyzed via fluorescence microscopy. Statistical analysis was performed using a two-sample *t*-test with unequal sample size and unequal variance, with asterisk indicating *P* < 0.005. Error bars show SEM of three independent experiments. (**C**) Kinetics of particle attachment quantified using flow cytometry. Where indicated, CCCP was added during incubation to reduce cell motility. As a negative control, biotin was added in excess to inhibit the cell-particle attachment. Statistical analysis, performed using a two-sample *t*-test with unequal sample size and unequal variance, showed that difference between all datasets was highly significant (*P* < 0.00001). Data are from six independent experiments. (**D**) Exemplary trajectories of bacteriabots with one, two and three attached WT *E. coli* cells, analyzed using time-lapse fluorescence microscopy. Acquisition time of depicted trajectories was 6.5 s (panel I), 7.4 s (panel II), 13 s (panel III), and 5.4 s (panel IV). Scale bar: 8 µm.

We next demonstrated that *E. coli* expressing Ag43-BAP could be attached to streptavidin-functionalized polymethyl methacrylate (PMMA) microparticles to form bacteriabots. Indeed, complexes between bacteria and microparticles could be visualized using either confocal fluorescence microscopy or scanning electron microscopy (SEM) (Fig. 2A). These images indicate preferential polar attachment of bacteria to microparticles, which was further confirmed by statistical analysis (Fig. 2B). Polar attachment was more pronounced for the wild-type cells compared to the non-flagellated strain that was used as a control, suggesting that it may be at least partly due to head-on collisions of swimming cells with the microparticles during the attachment process. However, preferential localization of the recombinant Ag43 to the *E. coli* cell pole might also contribute to polar attachment (Supplementary Fig. S1), since significant polar preference was observed even for the non-flagellated cells.

To better understand the importance of motility for attachment, we compared the attachment kinetics of motile and non-motile bacteria using flow cytometry to distinguish microparticle-attached cells from free cells and microparticles (Supplementary Fig. S3). The attachment of wild-type cells was rapid, reaching half-maximal value at ~6 min of incubation and approaching saturation after 20 min, when free particles became depleted (Fig. 2C). Excess of free biotin in the incubation reaction completely inhibited attachment, confirming specificity of bacteria-particle interactions. In contrast, the attachment of non-motile cells lacking flagella proceeded much slower. This difference was apparently due to the lack of motility, and not due to unspecific adhesion of cells via flagella^47^, because decreasing the swimming speed of wild-type *E. coli* by dissipating the proton motive force with the help of carbonylcyanide-m-chlorophenylhydrazone (CCCP) resulted in an even more pronounced decrease in the attachment rate. Expectedly, the effect of CCCP on the attachment rate of non-motile cells was minor, but the overall better attachment of the CCCP-treated non-flagellated cells compared to the wild type suggests that in absence of motility flagella might partly hinder the biotin-streptavidin mediated cell-particle attachment. Altogether, these data clearly show that motility promotes (polar) attachment, primarily through heads-on collisions of bacteria with microparticles.

Next, we characterized the dependence of bacteriabot movement on the number of attached bacteria (Fig. 2D and Supplementary Movie 1–5). We observed that in cases, when only one cell was attached to a microparticle, bacteria almost exclusively pulled the microparticle (Fig. 2D panel I and Supplementary Movie 1). For cases, when two bacteria were attached to the same particle so that their long cell axes aligned, the swimming behavior was similar to the particle pulled by a single cell, but the overall movement was markedly slower (Fig. 2D panel II and Supplementary Movie 2). This was also observed in very rare cases where two particles were aligned alternating between tree cells (Supplementary Movie 3). However, when the axes of the two cells were not aligned (Fig. 2D panel III and Supplementary Movie 4) or when more than two cells were attached to a microparticle (Fig. 2D panel IV and Supplementary Movie 5), the swimming behavior was largely compromised, with very little processive motion, likely due to the misalignment of the forces exerted by individual bacteria on the same particle. Thus, the simplest constellation consisting of one bacterial cell per microparticle provides the most efficient and fastest particle propulsion. In subsequent bacteriabot fabrication, we used a particle to cell mixing ratio of 1:30 at which the majority of observed bacteriabots consisted of one bacterial cell per particle, with the highest number and swimming speed of motile bacteriabots (Supplementary Fig. S4).

### Dependence of bacteriabot motility on particle size and cell length

**Figure 3:**
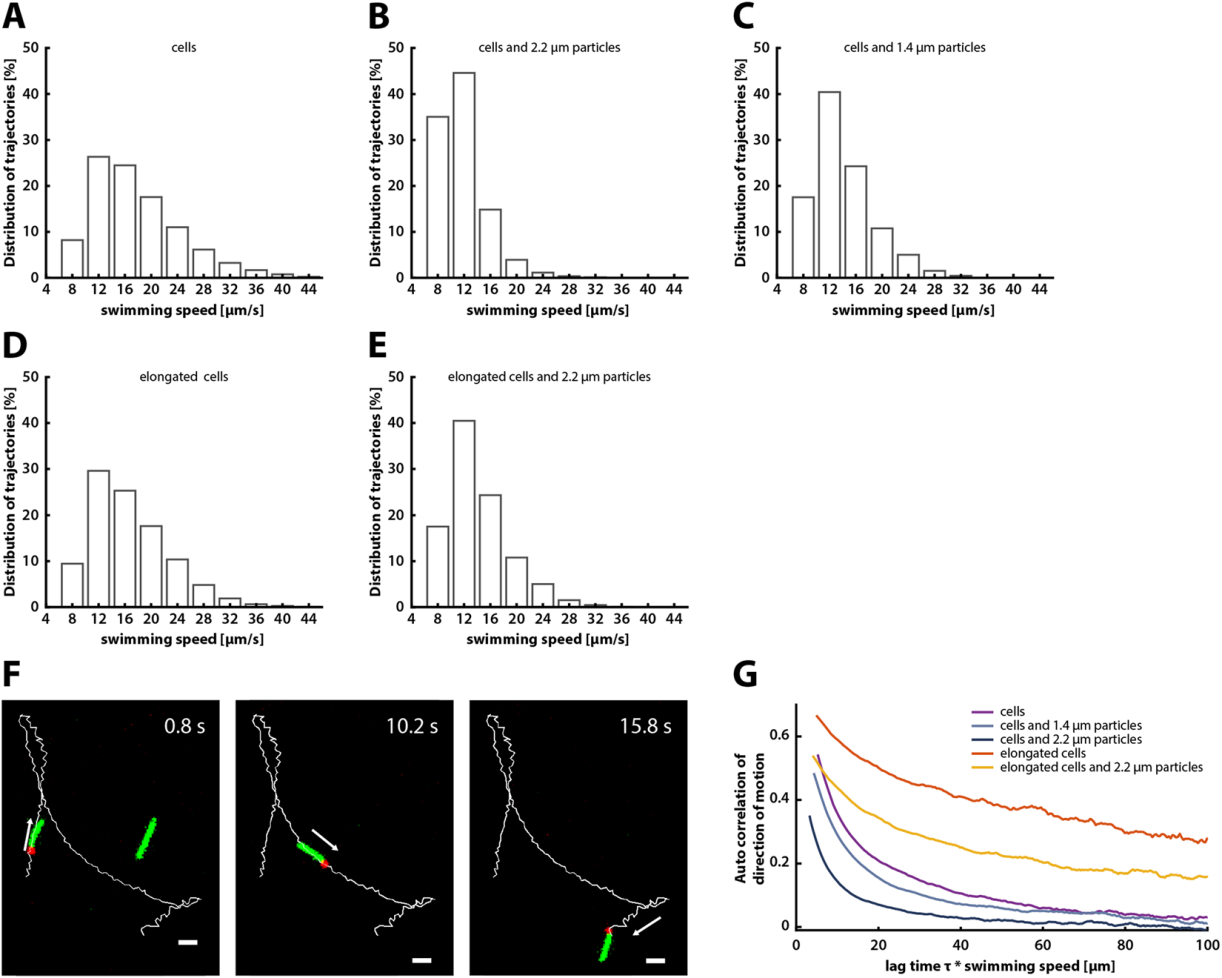
Motility of bacteriabots based on normal and elongated cells. (**A**–**E**) Distribution of swimming speed within populations of normal or elongated *E. coli* cells, with or without PMMA microparticles, as indicated. Average swimming speed of individual cells was determined based on 2D trajectories recorded using fluorescence microscopy, as described in Methods. Data are from seven or more independent experiments. Numbers of analyzed trajectories were 109192, 30724, 44698, 10601, and 10744 for cells, cells with 2.2-µm particles, cells with 1.4-µm particles, elongated cells, and elongated cells with 1.4-µm particles, respectively (**F**) Examples of 2D trajectories of bacteriabots based on elongated *E. coli* cells. Scale bar: 8 µm. (**G**) Autocorrelation of direction of motion, calculated from the trajectory data as described in Methods.

To analyze the effects of particle attachment on bacterial motility, we tracked two-dimensional (2D) swimming trajectories of *E. coli* cells that were either free swimming (Fig. 3A) or attached to 1.4-µm or 2.2-µm diameter particles (Fig. 3B,C). For consistency reasons, the measurements were performed within 30 to 90 min after placing the sample into the observation chamber. Free-swimming *E. coli* had a mean swimming speed of 15.71 ± 0.02 µm/s (±SEM), which was reduced to 12.83 ± 0.02 µm/s upon attachment of 1.4-µm particles and to 9.76 ± 0.02 µm/s upon attachment of 2.2-µm particles (Fig. 3B,C). Even more pronounced reduction was visible in the fraction of bacteria with the highest swimming speed (>20 µm/s) (Fig. 3A–C). Such reduction of the cell swimming speed is consistent with the increase in the rotational and translational friction coefficients because of the addition of a spherical particle, which could be computed by modeling flagellar propulsion using resistive force theory^48–50^ (Supplementary Fig. S7, Table S1 and Supplementary Text). Although cell motility in our assay decreased gradually over time (Supplementary Fig. S5), this decrease was slow and similar for free swimming cells and bacteriabots, indicating that particle attachment has no negative effect on the energy state of the cell.

As stated earlier, bacteriabots need to maintain their function in biological fluids to be of use for biomedical applications. One distinct property of biological fluids is their higher viscosity, up to 5 mPa·s in case of blood^51^ compared to <1 mPa·s for water. Therefore, we tested motility of bacteriabots when the viscosity of the medium was raised above 4 mPa·s by addition of methyl cellulose. The speed of both free swimming cells and bacteriabots was unchanged or even slightly increased at this higher viscosity (Supplementary Fig. S6A,B), which could be again explained by the resistive force theory (Supplementary Text).

How could the swimming properties of the *E. coli*-based bacteriabots be enhanced? We hypothesized that elongated *E. coli* cells would be less affected by the particle attachment. *E. coli* cells can be easily artificially elongated by growing them in presence of cephalexin, a β-lactam antibiotic that reversibly blocks cell division^52,53^, with the duration of cephalexin treatment determining the cell length. Such elongated *E. coli* cells are known to have more, and possibly also longer, flagella, although the increase in the number of flagella might not be linearly proportional to the increase in the cell length^49^, and they are able to perform chemotaxis^54^. We thus expected that – at a given particle size – the cephalexin-treated cells have a more favorable balance between the size of the cell body (and the number of flagella) and the particle size. Our calculations indeed predicted smaller reduction of the swimming speed for elongated cells upon attachment of the same-sized particle (Supplementary Fig. S7 and Supplementary Text).

Cells treated with cephalexin for 60 min, with an average length of approximately 9 µm, had a mean swimming speed of 14.66 ± 0.06 µm/s, similar to the speed of normal cells with an average size of approximately 3 µm (Fig. 3D). However, consistent with our prediction, particle attachment had much less effect on the swimming speed of elongated cells, which only decreased to 12.08 ± 0.04 µm/s for 2.2-µm particles (Fig. 3E). Similarly, the fraction of fast-swimming cells was also little affected.

Furthermore, the mode of microparticle propulsion was apparently different between normal and elongated cells. As mentioned above, normally-sized cells predominantly pulled the microparticles (Supplementary Movie 1), while pushing propulsion was apparently unstable and almost immediately reversed by a tumble. In contrast, elongated cells could apparently mediate both pulling and pushing propulsion (Fig. 3F and Supplementary Movie 6). The efficiency of the two modes of propulsion by the elongated cells was nearly equal, with pulling population being observed in 46% of cases, and the mean swimming speed during pulling and pushing runs being 14.9 ± 3.0 µm/s and 18.1 ± 5.2 µm/s, respectively. In a further contrast to particle propulsion by normally-sized cells, particle-loaded elongated cells changed their swimming direction by stopping and reversing their swimming orientation rather than by tumbling (Fig. 3F and Supplementary Movie 6), in agreement with previous studies^49,54^.

To further investigate how the swimming dynamics of normal and elongated bacteria was altered by an attached microparticle, we determined the time autocorrelation of direction of motion as well as the mean square displacement (MSD) of bacteria with and without attached microparticles. The autocorrelation of directional motion for elongated cells was decaying much slower with the distance than for normal cells (Fig. 3G), and their MSD curves (Supplementary Fig. S8) indicated more efficient, superdiffusive spreading. Both of these observations could be explained by the lower reorientation per change of direction for elongated cells and by the higher persistence in their direction of motion during runs (i.e., lower rotational diffusion) (Fig. 3F and Supplementary Fig. S9). The attachment of particles led to decreased autocorrelation of direction and persistence for both normal and elongated cells, suggesting that an attached particle increases the reorientation of the cell, either by causing larger reorientations during tumbles or reduced directional persistence during runs.

### Chemotaxis of bacteriabots

**Figure 4:**
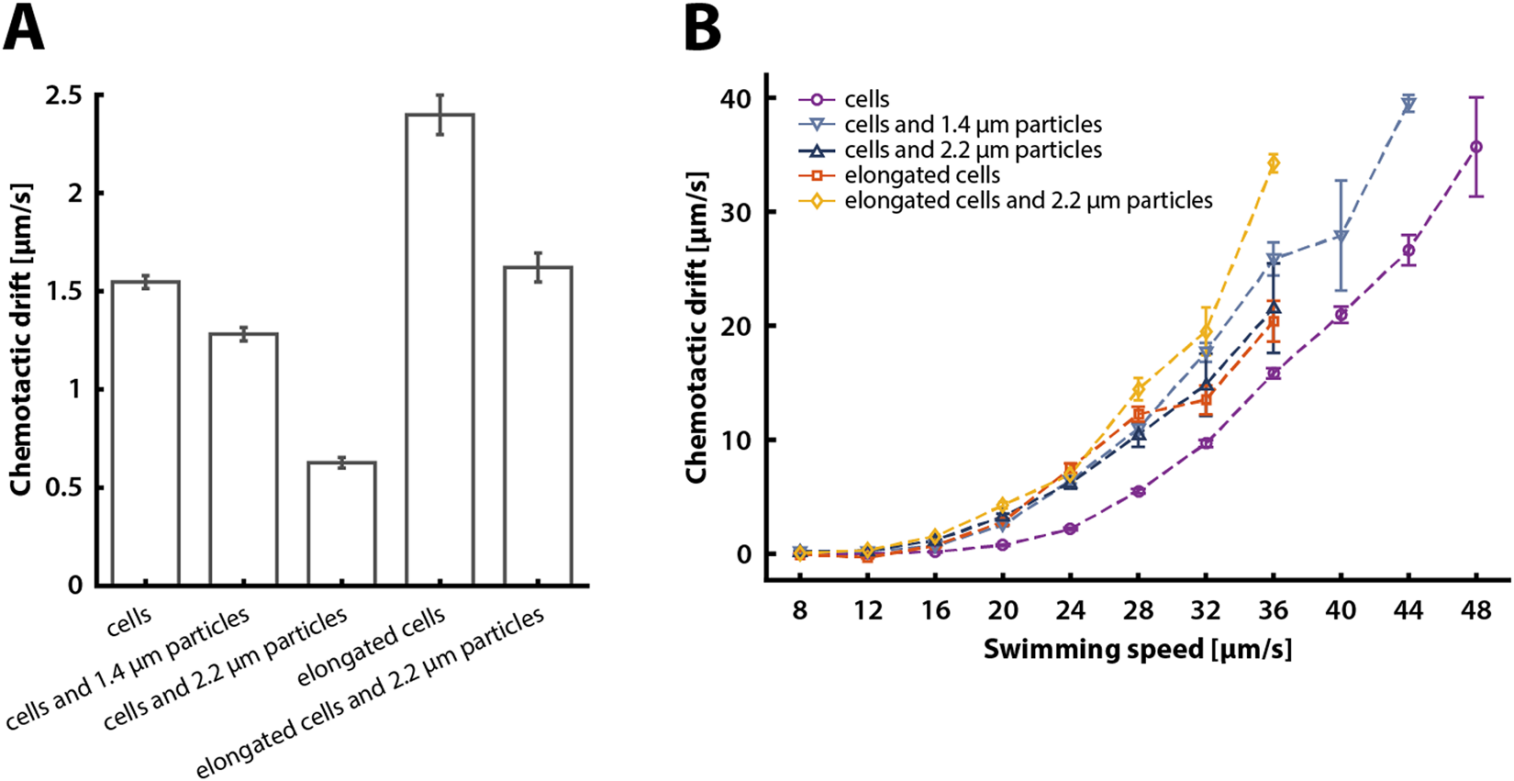
The chemotactic drift of free-swimming cells and bacteriabots. (**A**,**B**) Mean chemotactic drift (**A**) and chemotactic drift as a function of the swimming speed (**B**) were calculated from individual 2D trajectories of cells with or without particles, as indicated (see Methods for details). Statistical analysis in (**A**) was performed using a two-sample *t*-test with unequal sample size and unequal variance, yielding highly significant differences between all datasets (*P* < 0.00001). Error bars show SEM from seven or more independent experiments. Numbers of analyzed trajectories were 109192, 44698, 30724, 10601, and 10744 for cells, cells with 1.4-µm particles, cells with 2.2-µm particles, elongated cells, and elongated cells with 1.4-µm particles, respectively.

Finally, to investigate the capability of bacteriabots based on normal or elongated cells to perform chemotaxis, we monitored their behavior in the presence of a chemical gradient formed in a microscopic channel (see Methods). For normal free-swimming bacteria, the mean chemotactic drift in a linear gradient of 0 to 200 µM α-methyl-DL-aspartate (MeAsp) over 2 mm channel length was 1.55 ± 0.03 µm/s (Fig. 4A), within the range of previously reported values^55^. The chemotactic drift decreased strongly when normal bacteria were attached to microparticles, particularly pronounced for larger 2.2-µm diameter particles. This decrease was apparently due to the lower swimming speed of bacteriabots, since plotting the chemotactic drift as a function of the swimming speed for individual cells showed comparable – and even better – drift of bacteriabots at a given speed compared to free cells (Fig. 4B). Notably, the observed dependence of chemotactic drift on swimming speed was unaffected by an increased medium viscosity (Supplementary Fig. S6C,D). Overall, the dependence of the chemotactic drift on the swimming speed was very steep, scaling as ${v}_{0}^{\alpha }$ with *α* ranging from 2.5 to 3.4 (Supplementary Fig. S10A), which is markedly steeper than *α* = 2 expected from the general theory of bacterial chemotactic motion^24^ (Supplementary Fig. S10B). This is likely explained by other factors that contribute to the chemotactic drift being a function of swimming speed, such as the tumbling angle^56^, or signal amplification by the chemotaxis pathway^24^, which depends on the expression of chemotaxis and flagellar genes, and therefore correlates with the swimming speed.

Interestingly, elongated cells showed nearly the same dependence of the chemotactic drift on swimming speed with and without attached particles, which was higher than for free-swimming normal cells but comparable to that of the normal cells carrying particles. Consistently, the mean chemotactic bias (the chemotactic drift normalized by the squared swimming speed) was similar for bacteriabots and elongated cells and lower for free-swimming normal cells (Supplementary Fig. S11). These results suggest that the swimming speed is by far the major determinant of the bacteriabot’s capability to perform chemotaxis, irrespective of the cell length, medium viscosity or cargo attachment, and they show that cell elongation can enhance chemotaxis of bacteriabots.

## Discussion

There is an increasing interest in various applications of bacteria to deliver microscopic cargos, and several studies have shown that such bacteria-powered microswimmers can be principally used to move cargo in environmental gradients^6,15–19^. However, the applicability of bacteriabots remained limited, partly due to the lack of protocols for specific and fast loading of cargo, but also because mobility and tactic movement of bacteriabots were not well understood.

In this study, we developed and characterized a system for efficient generation of bacteriabots via biotin-streptavidin interaction. Although this interaction was already used to construct bacteriabots^7,17,21^, previous biotin functionalization of the bacterial cell surface required time consuming preparations with either biotin-conjugated antibodies targeting outer membrane proteins or the lipopolysaccharide^15^, or a chemical modification of the surface via biotin-NHS esters^35^. Because bacterial flagella are highly fragile, multistep preparation procedures can largely decrease the motility of the cell (due to the exposure to chemicals and shear forces) and thus decrease the applicability of the resulting bacteriabots. Instead, we engineered *E. coli* to display biotin on its surface using autotransporter Ag43 that carries the biotinylation peptide BAP. This autonomous biotinylation of the cell surface via Ag43-BAP is of great value in applications where rapid and stable cell-surface attachment is needed. Besides, the engendered Ag43-BAP potentially allows introduction of additional features to the display system, such as a protease restriction site for a targeted release of the attached particle. The extent of the biotin functionalization of the cell surface in this strain could be modulated by varying the expression level of Ag43-BAP and by the availability of biotin in the medium. Yet another advantage of labeling mediated by Ag43 is that its N-terminal protrusion overcomes the LPS layer of the bacterial cell surface, which otherwise limits the accessibility of displayed peptides^42,43,57,58^. As Ag43 can be expressed and exposed on the surface in a brought range of gram negative bacteria, including *Salmonella enterica, Burkholderia cepacia, Pseudomonas aeruginosa, Pseudomonas fluorescens, Klebsiella pneumoniae, Enterobacter cloacae* and *Serratia liquefaciens*^40,59^, this display system is principally compatible with various biomedical applications of bacteriabots based on these species^60,61^.

The attachment of particles using this Ag43-BAP labeling approach showed high efficiency and yield, with most particles loaded on the wild-type cells already within ten minutes of incubation. We observed that the attachment was strongly enhanced by cell motility, suggesting that the rather straight motion of motile cells promotes heads-on collision and attachment to the particle. The heads-on collisions between motile cells and particles could also partly explain strong bias towards polar attachment observed in the wild-type cells, although the enrichment of Ag43 at the cell pole may further contribute to this bias. Such polar attachment of cargo is beneficial for the movement of bacteriabots, because it ensures alignment of the center of mass with the propulsion direction and stabilizes the ballistic motion of the bacteriabot.

The observed effects of the particle attachment on motility and chemotaxis further allowed us to draw several general conclusions about the fabrication and use of bacteriabots. First, we could demonstrate that attachment of multiple bacteria to the same particle hinders particle motion rather than facilitating it. This effect may be partly due to the misalignment between the axes of individual cells, and thus of generated propulsion forces. However, even in rare cases when the axes of multiple cells were aligned, the mobility of the attached particle was lower than upon attachment of a single cell, likely because of the steric hindrance of the flagella bundle but also due to uncoordinated switching of flagella in individual cells. Thus, random attachment of multiple bacteria to the same microparticle may be generally undesirable for cargo loading, although in principle defined patterning of the microparticle might allow lateral alignment of cell bodies and therefore a better coordinated propulsion^62^.

We further observed that the chemotactic capability of bacteriabots was exclusively limited by their swimming speed. In contrast, increased viscosity, as encountered in body fluids, had little effect on the swimming speed, and thus on chemotaxis. In general, the dependence of the chemotactic drift on the swimming speed was very steep, even steeper than expected from the theory of bacterial chemotactic motion^24^. The capability of both cells and bacteriabots to perform chemotaxis is thus extremely sensitive to their swimming speed, meaning that even minor speed reduction due to cargo loading would have substantial effect on the chemotactic efficiency. Consistent with that, although at similar swimming speed the efficiency of chemotactic drift of the bacteriabots was even higher than for regular-sized free-swimming *E. coli* cells, their mean chemotactic drift was largely reduced due to the slower movement of bacteriabots compared to free cells. This was particularly pronounced upon attachment of the larger, 2.2-µm, particles, but was also visible for smaller 1.4-µm particles.

Together, the steep dependences of the chemotactic drift on the swimming speed and of the swimming speed on the load imply that the effective size of cargo that can be carried by the chemotactic bacteriabots based on normal *E. coli* cells is limited to approximately 2 µm. However, we could circumvent this limitation by using *E. coli* cells that were elongated by treatment with cephalexin, an antibiotic blocking cell division. Such elongated cells have a mean swimming speed similar to that of normal cells, which could indicate that they possess longer flagella, although their increased number of flagella^36^ could also play some role. Nevertheless, the chemotactic drift in linear gradients apparently increased upon cell elongation, likely due to the higher persistence length of these elongated cells, in consensus with the previous work^36^. Most importantly, the swimming speed of elongated cells – and therefore their chemotaxis – was much less affected by the particle attachment. Our work thus suggests that elongated cells are superior compared to normal *E. coli* cells for bacteriabot fabrication. This seems to be partly due to the more favorable relation between the size and number of flagella in these cells relative to the size of the cargo particle. Besides, high motion persistence, lower rotational diffusion and the shifted center of mass of the bacteriabot towards the propelling elongated cell probably allows for the observed stable pulling and pushing of the particle.

## Methods

### Strain construction and growth conditions

All *E. coli* strains were derived from MG1655 (defined here as the wild type; WT)^63^. Plasmid pOS239 was derived from pREP4 (Qiagen, Germany), and it includes the gene for the transcriptional regulator AraC and the arabinose-inducible promotor pBAD driving the expression of EGFP. pOS233 and pOS200 were derived from pSB1A3 (Registry of Standard Biological Parts, USA) and they include an IPTG-inducible T5 promotor^64^ and the 1–170 nt and 313–2989 nt of the coding region of *E. coli* antigen 43 (Ag43). In pOS233 (Ag43-BAP) the nucleotides 171–312 of Ag43 were replaced by the coding sequences of the FLAG epitope and biotin acceptor peptide (BAP), whereas the BAP was absent in pOS200 (Ag43-FLAG).

Cells were grown at 34 °C and 275 rpm in tryptone broth (TB; 1% w/v tryptone, 0.5% w/v NaCl, pH 7.0), supplemented with the appropriate antibiotics (100 µg/ml ampicillin and 50 µg/ml kanamycin) and with or without the addition of 1 µM D(+) biotin. The cell growth was monitored with a Tecan Infinite M1000 Pro Microplate Reader (Tecan, Switzerland). Cell cultures were inoculated with a 1:100 diluted overnight culture. After 2 hours of cultivation, the expression of recombinant Ag43 and EGFP was induced by the addition of 100 µM isopropyl-β-D-thiogalactopyranoside (IPTG) and 0.005% w/v L(+) arabinose, respectively. Cells were cultivated for additional 2 to 2.5 hours until an OD_600_ of 0.6 was reached. Where indicated, 10 μg/ml cephalexin was added to the culture for 60 min before harvesting cells. Cells were washed and resuspended in motility buffer (10 mM KPO_4_, 0.1 mM EDTA, 67 mM NaCl, pH 7), supplemented with 1% w/v glucose and 0.5% w/v BSA.

### Bacteriabot fabrication

Cells were prepared as described above and resuspended to a final OD_600_ of 0.0375 in motility buffer. The cell suspension was mixed in a ratio of 1:30 (in numbers) with 1.4-µm or 2.2-µm Red4 streptavidin PMMA particles (1.5 × 10^3^ particles/µl; PolyAn, Berlin) and incubated for 20 min at room temperature. The kinetics of cell-particle attachment was analyzed using a BD LSRFortessa SORP cell analyzer (BD Biosciences, Germany). Where indicated, carbonylcyanide-m-chlorophenylhydrazone (CCCP; Sigma Aldrich, Germany) was added during incubation.

### Immuno- and NeutrAvidin-biotin staining

The detection of recombinant Ag43 and of the surface-displayed biotin was performed via immunostaining and biotin staining. For immunostaining, the cell suspension (OD_600_ of 2.0) was incubated with 1 µg/ml monoclonal mouse anti-FLAG^®^ M2 antibody (Sigma Aldrich, Germany) or 1 µg/ml monoclonal mouse anti-biotin BTN.4 antibody (Thermo Fisher, Germany) for 1 hour at room temperature. After washing three times, cells were resuspended in motility buffer and incubated with 1 µg/ml anti-mouse goat IgG conjugated to Cy3 (Thermo Fisher, Germany) for 1 hour at room temperature. For NeutrAvidin-biotin staining, the cell suspension (OD_600_ of 2.0) was incubated with 1 µg/ml NeutrAvidin conjugated to DyLight™ 488 (Thermo Fisher, Germany) for 45 min at room temperature. The fluorescence images were acquired at 488 and 587 nm for DyLight™ 488 and Cy3 stained cells, respectively, using a Nikon Eclipse Ti-U fluorescence microscope (Nikon Instrument, Japan) with 100x objective and Zyla 4.2 Plus sCMOS camera (Andor Technology Ltd, UK). Quantification of biotinylation was performed using flow cytometry as above. Extracellular biotinylation of Ag43-BAP was performed by incubating the cell suspension (OD_600_ of 2.0) with 0.1 mg/ml BirA (Avidity, USA) in motility buffer supplemented with 10 mM ATP, 10 mM MgOAc and 50 µM d-biotin for 1 hour at 30 °C. Samples were subsequently washed tree times with motility buffer and stained via NeutrAvidin, DyLight™ 488 conjugate.

### Widefield fluorescence microscopy

The fluorescence imaging of cells and particles was performed at 488 nm using a Nikon Eclipse Ti-U fluorescence microscope (Nikon Instrument, Japan) using a 40x objective and a dual emission image beam splitter with 525/50 nm and 647/57 nm mounted emission filters (Optosplit II; Cairn Research, UK) connected to an iXon3 897 EMCCD camera (Andor Technology Ltd, UK) at 10 frames per second. This setup enabled dual-color time-lapse microscopy of green fluorescent cells and red fluorescent particles. From these data, the propulsion orientation (push or pull) of the cell and the respective swimming speed was determined. The same data set was used to analyze the particle attachment along the cell body, with a particle attached within the first fifth of the cell body being considered as polar attachment.

### Confocal laser scanning microscopy and scanning electron microscopy

The cell-particle attachment was visualized using a Zeiss Axio Observer Laser Scanning Microscope (LSM) 880 (Zeiss, Germany) using a 40x objective. Samples were illuminated using a 488 nm Argon and 561 nm DPSS laser. Z-stack projections were analyzed using the Zeiss ZEN System imaging software (Zeiss, Germany). Samples were also imaged using a Supra 55VP scanning electron microscope (Zeiss, Germany) using an accelerating voltage of 3 kV and an in-lens detector.

### Microfluidics and cell tracking in chemical gradients

The swimming behavior of cells and bacteriabots fabricated as described above was analyzed using a poly-di-methylsiloxane (PDMS) microfluidic device consisting of two large reservoirs connected by an observation chamber (2 mm in length and 1 mm in width), constructed by standard photolithography techniques as described previously^55,65^. The microfluidic chamber was filled with a solution containing 1 M lithium acetate and 1% w/v denatured BSA, preventing particles from adhering to its walls. After 15 min both reservoirs were filled with motility buffer containing cells or bacteriabots, where one reservoir contained additionally 200 µM α-methyl-DL-aspartate (MeAsp). A linear gradient of MeAsp formed across the observation chamber within an hour^65^. Fluorescence imaging of cell and bacteriabots (particles) was performed at 488 nm and 587 nm, respectively, using a Nikon Eclipse Ti-U fluorescence microscope (Nikon Instrument, Japan) with a 40x objective and a Zyla 4.2 Plus sCMOS camera (Andor Technology Ltd, UK) at 33 frames per second.

Cells and bacteriabots were tracked using Fiji^66^, with a custom-written particle-tracking plugin using centroid localization algorithm to detect particles and identification of the closest particle in next frame for trajectory linking^67^. The instantaneous velocity *v*_*i*_(*t*) (in 2D) of the object I was also determined by linear fit of the trajectory position *r*_*i*_(*t*) on a 10-frames long sliding window around time point *t*. Further processing via MATLAB (MathWorks, USA) yielded the population-averaged swimming speed *v*_0_, chemotactic drift *v*_*ch*_, chemotactic bias $\frac{{{\boldsymbol{v}}}_{ch}}{{v}_{0}^{2}}$, the mean square displacement $\langle {\rm{\Delta }}{r}^{2}(t)\rangle$ and the auto-correlation of direction of motion *C*_*v*_(*t*), as:

1 $${v}_{0}=\sum _{i}\sum _{t}^{{T}_{i}}|\,{{\boldsymbol{v}}}_{i(t)}|/\sum _{i}{T}_{i}$$

2 $${{\boldsymbol{v}}}_{ch}=\sum _{i}\sum _{t}^{{T}_{i}}{\boldsymbol{v}}\_i(t)/\sum _{i}{T}_{i}$$

3 $$\langle {\rm{\Delta }}{r}^{2}(t)\rangle =\sum _{i}\sum _{t\mbox{'}}^{{T}_{i}-t}{({{\boldsymbol{r}}}_{i}(t+t\mbox{'})-{{\boldsymbol{r}}}_{i}(t\mbox{'}))}^{2}/\sum _{i}({T}_{i}-t)$$

4 $${C}_{v}(t)=\sum _{i}\sum _{t\mbox{'}}^{{T}_{i}-t}\,\frac{{{\boldsymbol{v}}}_{i}(t+t\mbox{'})\cdot {{\boldsymbol{v}}}_{i}(t\mbox{'})}{\overline{{v}_{i}^{2}}}/\sum _{i}({T}_{i}-t)$$

Here, *T*_*i*_ is the duration of trajectory *i*. Only trajectories longer than 20 frames and for which the average swimming speed $\overline{{v}_{i}}=\frac{{\sum }_{t}^{{T}_{i}}|\,{{\boldsymbol{v}}}_{i}(t)|}{{T}_{i}}$ was higher than 5 µm/s were considered, to avoid artifacts arising from spurious detections and from non-swimming objects, respectively. Furthermore, trajectories were sorted according to their average swimming speed to plot data in Fig. 4B and Supplementary Fig. S11 using Equations [1, 2] for each subset of trajectories. In Supplementary Fig. S8, the mean-square displacement of each trajectory was calculated using Equation [3] and normalized by the respective ${{\boldsymbol{v}}}_{0}^{2}.$ Auto-correlation of direction of motion in Fig. 3D was calculated for all trajectories using the Equation [4], lag times were multiplied by the respective swimming speed *v*_0_. Persistence length $\xi$ was computed by fitting the autocorrelation according to ${C}_{v}(t)={C}_{0}{e}^{-{v}_{0}t/\xi }+{A}_{0}$.

## Electronic supplementary material

Supplementary materials

Supplementary Movie 1. Bacteriabot motility: one bacterium attached to one particle

Supplementary Movie 2. Bacteriabot motility: two bacteria attached to one particle (aligned)

Supplementary Movie 3. Bacteriabot motility: three bacteria attached to two particles (aligned)

Supplementary Movie 4. Bacteriabot motility: two bacteria attached to one particle (non-aligned)

Supplementary Movie 5. Bacteriabot motility: three bacteria attached to one particle (non-aligned)

Supplementary Movie 6. Bacteriabot motility: one elongated bacterium attached to one particle
