# Supplementary material for: Motility and chemotaxis of bacteria-driven microswimmers fabricated using antigen 43-mediated biotin display
Source: Sci Rep. 2018 Jun 28;8:9801. doi: 10.1038/s41598-018-28102-9 (PMC6023875; doi:10.1038/s41598-018-28102-9)
Supplement: Supplementary file 1 — Supplementary materials [file 41598_2018_28102_MOESM1_ESM.docx]

**Motility and chemotaxis of bacteria-driven microswimmers fabricated using antigen 43-mediated biotin display**

Oliver Schauer^1^, Babak Mostaghaci^2^, Remy Colin^1^, Daniel Hürtgen^1^, David Kraus^1^, Metin Sitti^2*^, Victor Sourjik^1*^

^1^Department of Systems and Synthetic Microbiology, Max Planck Institute for Terrestrial Microbiology & LOEWE Center for Synthetic Microbiology (SYNMIKRO), 35043 Marburg, Germany

^2^Physical Intelligence Department, Max Planck Institute for Intelligent Systems, 70569 Stuttgart, Germany

^*^Corresponding authors: Victor Sourjik, victor.sourjik@synmikro.mpi-marburg.mpg.de; Metin Sitti, sitti@is.mpg.de

# Supplemental Information

# Supporting Text

Calculation of swimming speed dependence on cargo using the resistive force theory for flagellar bundle

By rotating its corkscrew-shaped flagellar bundle, *E. coli* propels itself in water, which appears as an extremely viscous liquid at the cell’s length scale – *i.e.* the Reynolds number is low. In this situation, resistive force theory[^50^](#_ENREF_50) can be applied to compute the propulsion speed of the cell as a function of the geometries of the flagellum and the cell body as well as the characteristics of the flagellar motor.

Following the works of Purcell[^48^](#_ENREF_48), the symmetries of flows at low Reynolds number yield a linear set of relations between, on the one hand, the velocity ($v$) and rotation frequency ($\omega$) of the flagellar bundle and, on the other hand, the force ($F_{thrust}$) and torque ($N_{fl}$) it generates:

$$- F_{thrust} = A v - B \omega[5a]$$

$$N_{fl} = -B v + D \omega[5b]$$

Since no net force is exerted on the cell (or bacteriabot), these force and torque equate the viscous force and torque experienced by the cell body (with its attached bead when relevant), which also moves at speed $v$ and rotate at angular velocity $\Omega$.

$$F_{thrust} =A_{0} v [6a]$$

$$N_{fl} = - D_{0} \Omega[6b]$$

Here, $A, B, D$ and $A_{0}, D_{0}$ are the elements of the so-called propulsion matrices of respectively the flagellar bundle and the cell body. Notably, $B$ measures the conversion of rotation into translation (and vice-versa) thanks to the corkscrew shape of the bundle, and $A$ (resp. $A_{0}$) and $D$ (resp. $D_{0}$) are the translational and rotational friction coefficients of the bundle (resp. body). For the bundle modeled as a rigid helix, they can be calculated using resistive force theory[^50^](#_ENREF_50) and depend only on the geometry of the organelle and the viscosity of the fluid, as:

$$A = K_{n} l\sin\psi\left( \tan\psi+\frac{\gamma_{k}}{\tan\psi} \right) [7a]$$

$$B = K_{n} l R\sin\psi\left( 1-\gamma_{k} \right) [7b]$$

$$D = K_{n} l R^{2}\sin\psi\left( \frac{1}{\tan\psi} +\gamma_{k}\tan\psi\right) [7c]$$

with

$$\tan\psi= 2\pi\frac{R}{\lambda} [8]$$

$$K_{n}=\frac{4\pi\eta}{\ln\left( 1.8\frac{\lambda}{r} \right)+1/2} [9]$$

where $l$, $R$ and $\lambda$ are the helix length, radius and pitch respectively, $\psi$ is the pitch angle relative to the swimming axis, $r$ is the radius of the bundled filaments (composed of three or more 13 nm[^68^](#_ENREF_68) thick flagella) and $\gamma_{k}$ is the ratio of the tangential ($K_{t}$) to perpendicular ($K_{n}$) friction coefficients of the bundled filament (Supplementary Fig. S10B). All values for *E. coli* bundle used here are listed in Supplementary Table S1.

The cell body and its bead cargo are modeled by an equivalent cylindrical rod of length $L_{eff}$ and diameter $d_{eff}$ as follows:

$$L_{eff} = L_{c} + d_{b} [10a]$$

$$d_{eff} =\frac{L_{c}}{L_{eff}} d_{c} +\frac{d_{b}}{L_{eff}}d_{b} [10b]$$

with $L_{c}$ and $d_{c}$ indicating cell body length and diameter, and $d_{b}$ the bead diameter (Supplementary Fig. S10A). Defining the aspect ratio $p = L_{eff}/d_{eff}$, the friction coefficient of this equivalent rod is[^69^](#_ENREF_69):

$$A_{0}= 2\pi\eta\frac{L_{eff}}{\ln p-0.207+0.98/p}. [11]$$

Equations [5] and [6] can be solved to yield the swimming speed as a function of the motor rotation speed $\omega^{m} =\left| \Omega\right|+\left| \omega\right|$, considering that for *E. coli,* $D\ll D_{0}$ and $B^{2}\ll\left( A+A_{0} \right)D_{0}$,[^49^](#_ENREF_49):

$$v= B\frac{\omega^{m}}{\left( A + A_{0} \right)} . [12]$$

When cells are elongated or attached to beads, the friction coefficients $A_{0}$ and $D_{0}$ increase. From equation [12] we gather a first obvious effect of an increase of $A_{0}$ on swimming speed. The rotation speed $\omega^{m}$ could also change due to an increase of the total torque experienced by the motors. Indeed, the torque-rotation speed characteristic curve was measured for the *E. coli* motor[^70^](#_ENREF_70). In first approximation, the motor rotation speed is more-or-less constant, as long as the torque necessary to rotate the flagellum and its load ($N^{m}=\left| D\omega\right|+\left| D_{0}\Omega\right|$) stays below a saturation value, above which the motor can only generate a constant torque and thus rotates more slowly. We first assume that in all our experiments the load is below saturation and the rotation speed stays constant.

The predicted swimming speed was computed from equations [7], [10], [11] and [12]. Using the parameters displayed in Supplementary Table S1, a relatively good agreement was found with the experimental values (Supplementary Fig. S7B). Notably, the values of predicted swimming speed for different conditions ranked as the experimental ones. For the cephalexin-treated cells, it was necessary to assume that flagellar length increased to account for the relatively high swimming speed that was experimentally observed. Normal cells loaded with the 2.2-µm beads had a swimming speed lower than expected from the prediction, which could indicate that $\omega^{m}$ started to decrease because of a high load. Since $D_{0}$ scales as $L_{eff}^{3}$ [^69^](#_ENREF_69), the total load on the motors of cephalexin treated cells is expected to be even higher, but the increased flagella number should keep the torque per motor below saturation, explaining why a constant $\omega^{m}$ accounts for the swimming speeds in this case.

Note that the predicted swimming speed does not depend on the viscosity of the fluid. We indeed observed that the swimming speed was largely unaffected by raising the viscosity of the medium to ~4.3 mPa·s using methyl cellulose (Supplementary Fig. S5). It might have even increased slightly for the wild type cells, a phenomenon often reported[^71^](#_ENREF_71) and assumed to come from the viscoelastic properties of the fluid[^72^](#_ENREF_72), which is however beyond the scope of our model.

#
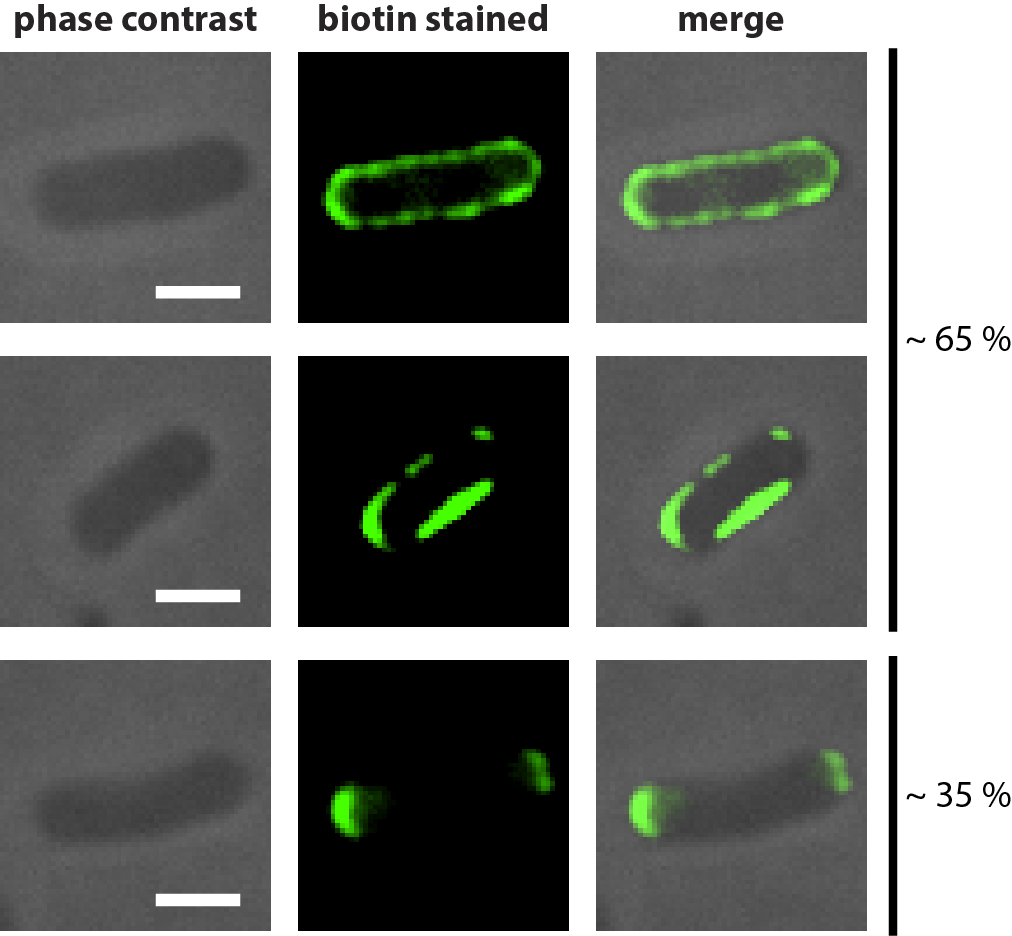


**Supplementary Figure 1. Localization pattern of biotin staining**. Fluorescence images of *E. coli* cells carrying Ag43-BAP that were incubated with NeutrAvidin-conjugated green dye. Scale bar: 2 µm.


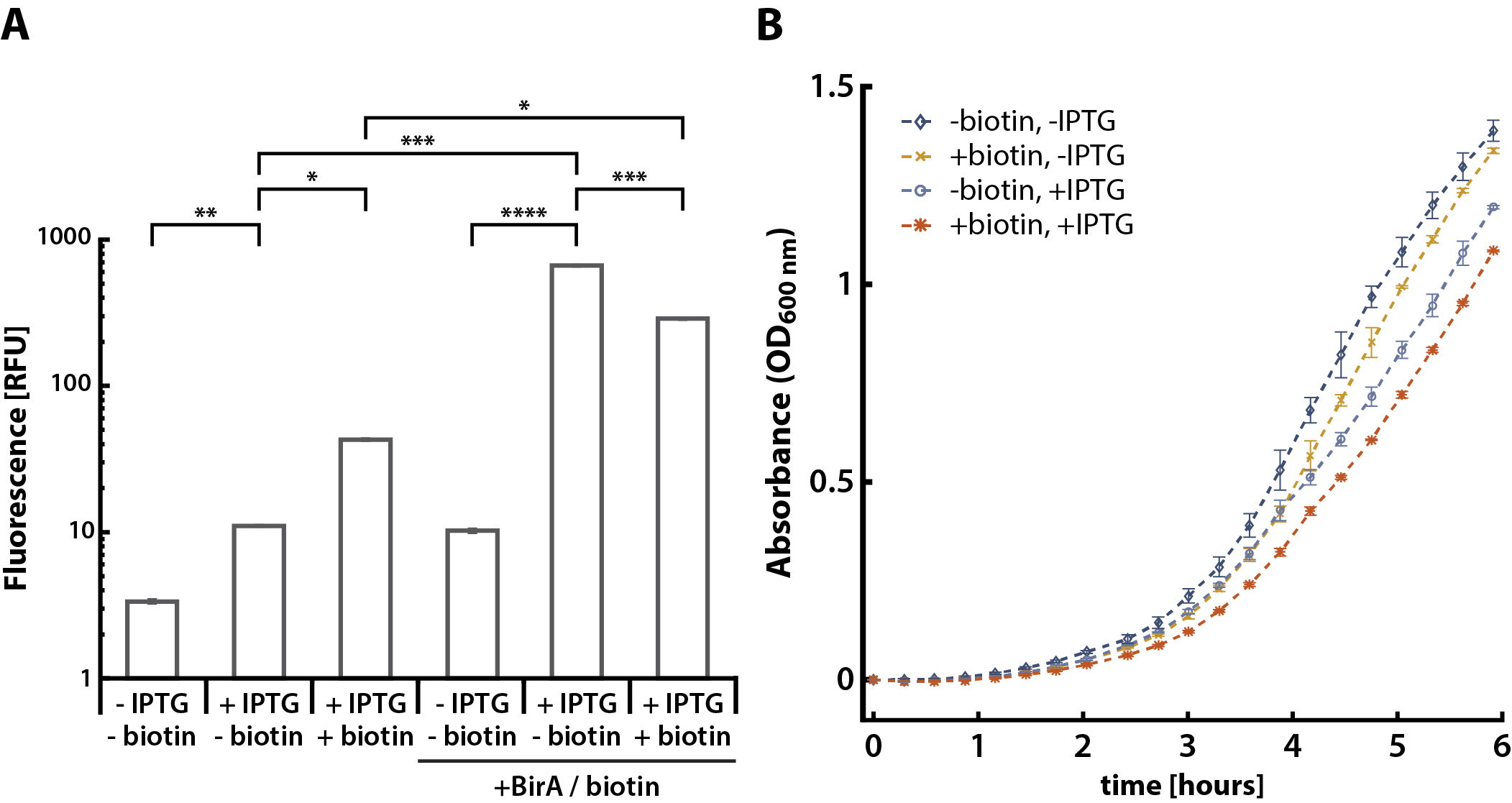


**Supplementary Figure 2. Quantification of biotinylated Ag43-BAP displayed on the cell surface of *E. coli*.** *E. coli* cells carrying a recombinant Ag43-BAP were grown with or without 1 µM biotin and/or 100 µM IPTG in the growth medium, as indicated. Cells were subsequently incubated with or without BirA / biotin. **(A)** Biotinylation was quantified by anti-biotin immunostaining measured via flow cytometry, as described in Methods. Statistical analysis was performed using a two-sample *t*-test with unequal sample size and unequal variance, with *P* < 0.001 (*), *P* < 0.0001 (**), *P* < 0.00001 (***), and *P* < 0.000001 (****). Data are from six independent experiments. **(B)** Growth curves in the various conditions were obtained from three independent experiments. Cell density was measured via absorbance at 600 nm. Error bars show SEM.


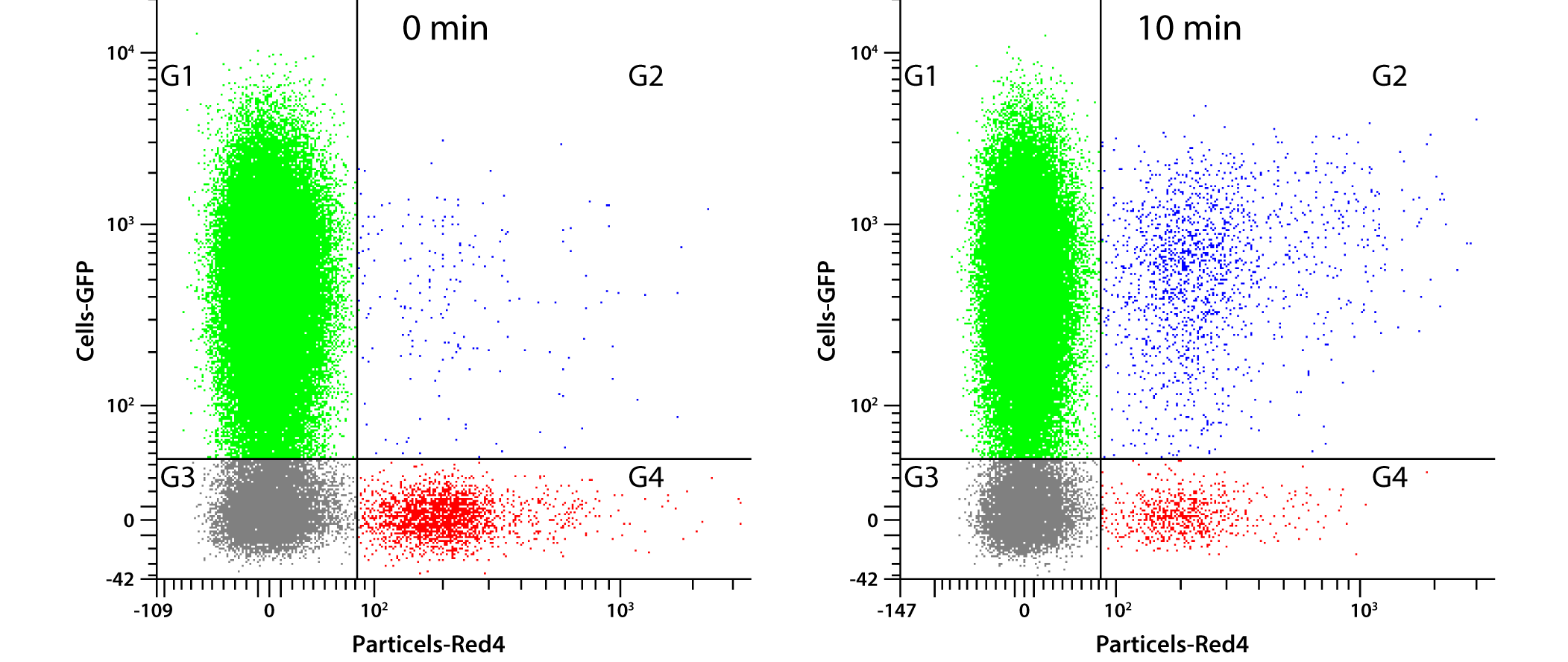


**Supplementary Figure 3. Example of flow cytometry analysis of microparticle attachment (see Fig. 2A).** Cells expressing Ag43-BAP and GFP (green) were incubated with 2.2-µm Red4 streptavidin PMMA particles (red), and samples right after mixing (0 min) and after 10 min of incubation were measured using flow cytometry. G1 and G4 correspond to free cells and particles, respectively; G2 to cells attached to particles; and G3 to cell debris / non-fluorescent cells.


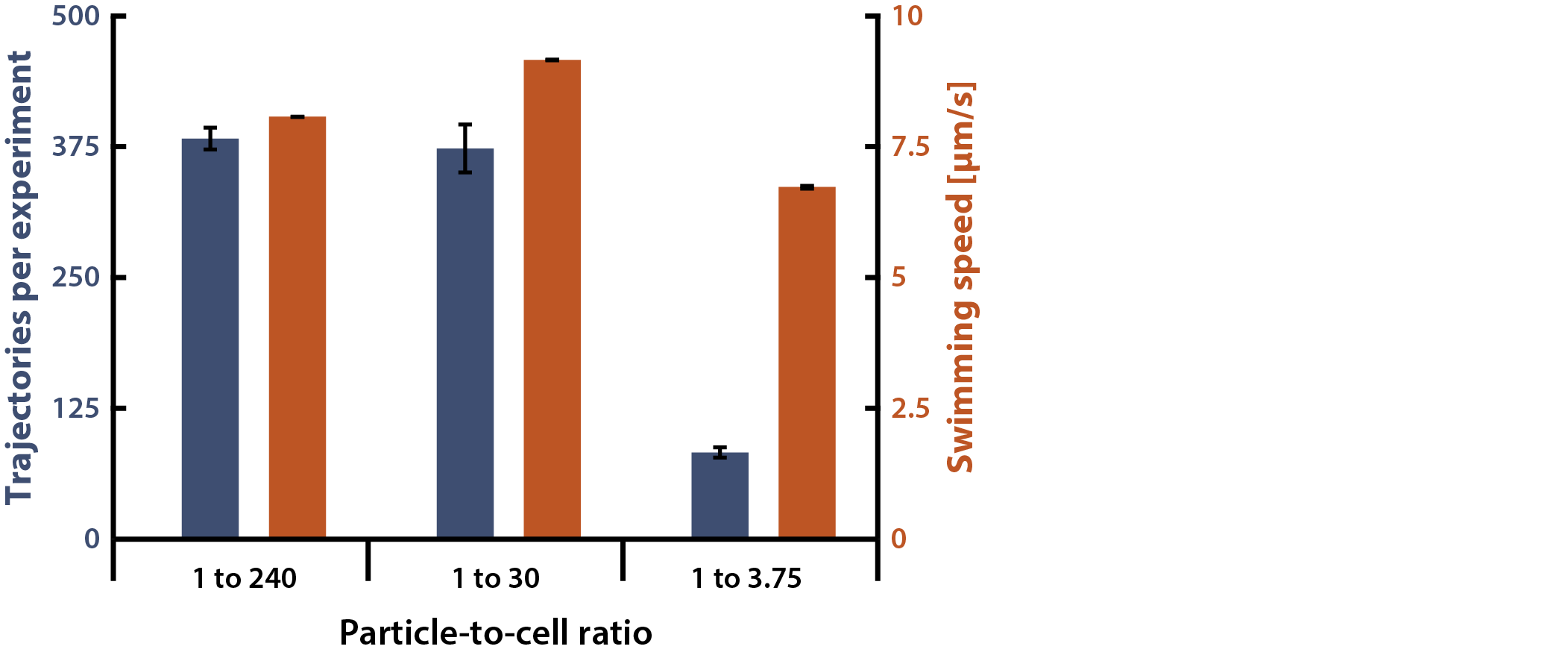


**Supplementary Figure 4. Fabrication of bacteriabots at various particle-to-cell ratios.** *E. coli* cells carrying the recombinant Ag43-BAP and an inducible GFP construct were incubated for 20 min with streptavidin-coated 2.2-µm PMMA particles, with a particle-to-cell mixing ratio of 1:240, 1:30 and 1:3.75, as indicated. Number of trajectories and mean chemotactic drift were calculated from individual 2D trajectories of the particles. Error bars show STD of seven independent experiments.
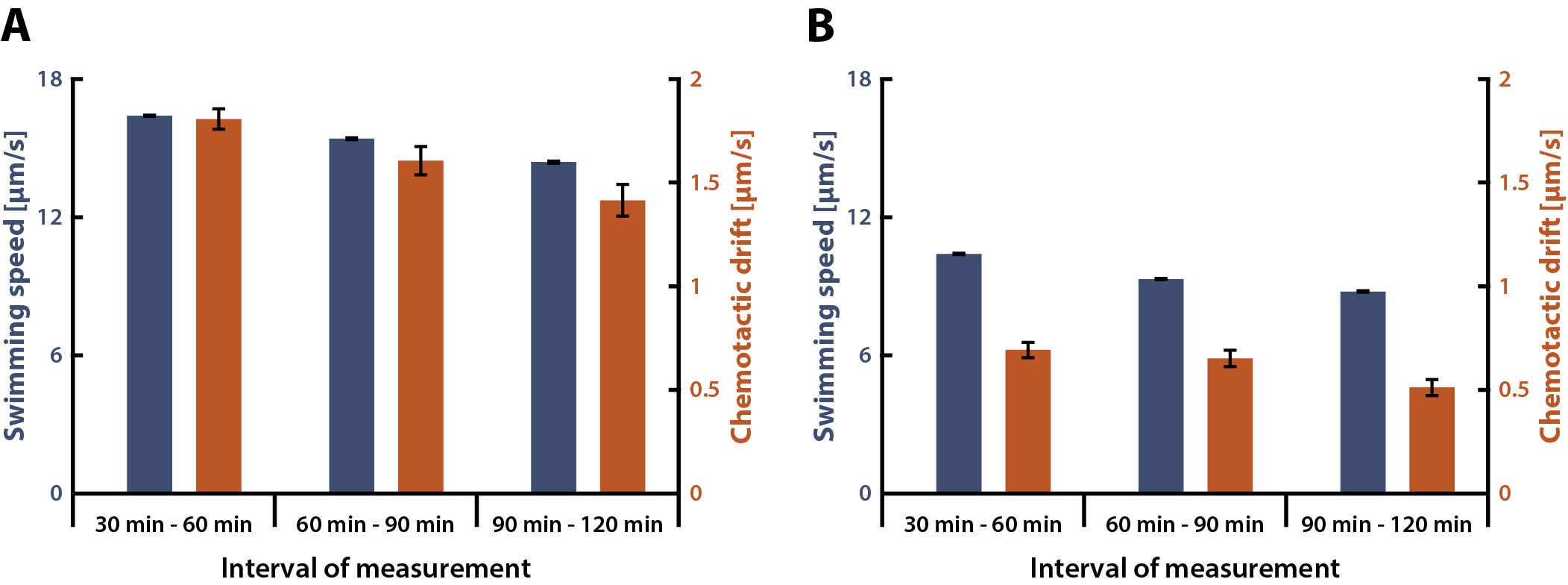


**Supplementary Figure 5.** **The swimming speed and chemotactic drift of free-swimming cells and bacteriabots at different time intervals.** **(A, B)** Mean swimming speed and chemotactic drift were calculated from individual 2D trajectories (see Methods for details) of cells without (A) or with 2.2-µm particles (B). Measurements were performed at different time intervals (as indicated) after placing the sample into the observation chamber. Error bars show STD of four independent experiments. Numbers of analyzed trajectories for the three indicated time intervals were 53901, 25954, 17058 for free-swimming cells and 20165, 13343, 8674 for cells with 2.2-µm particles.


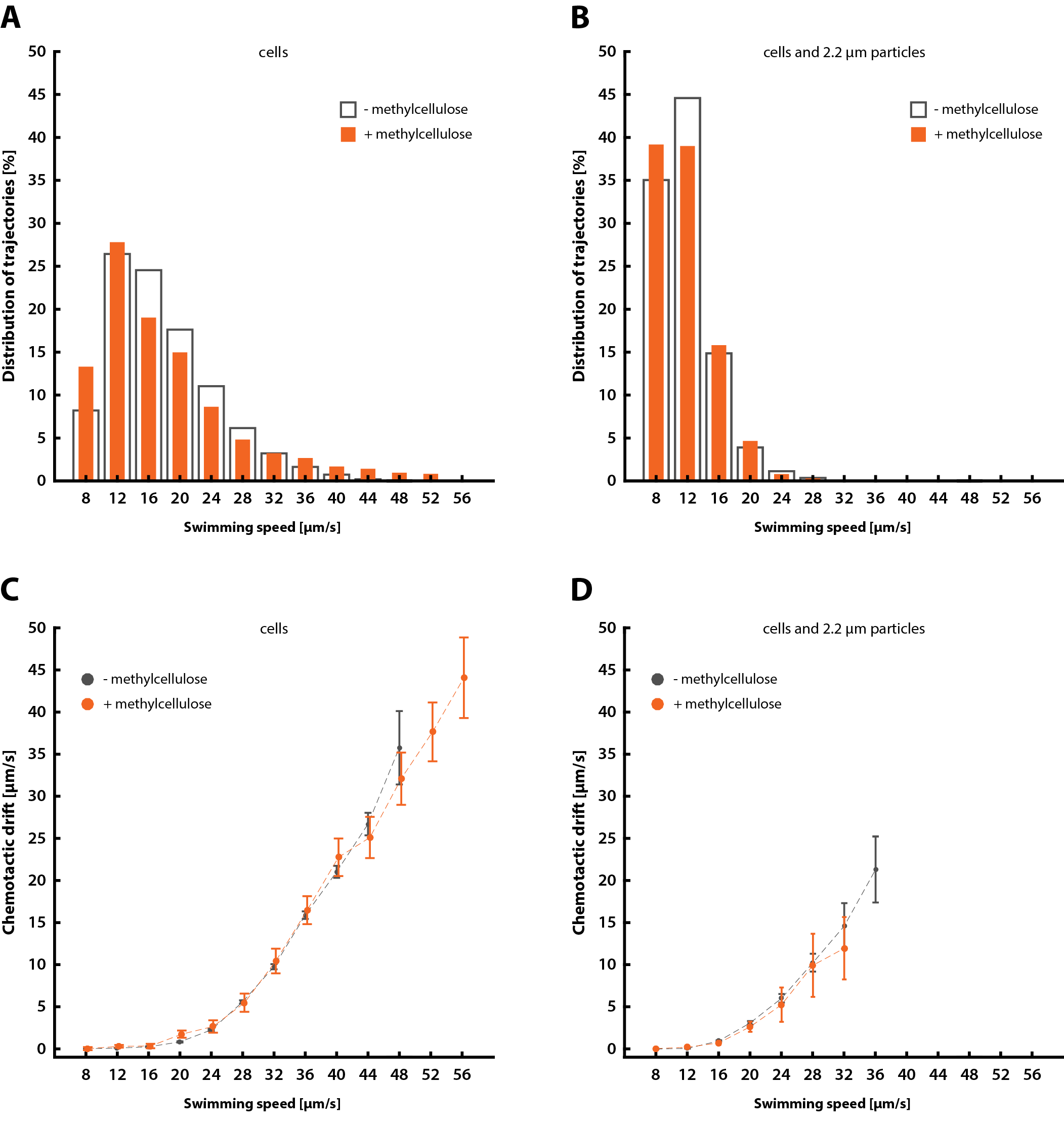
 **Supplementary Figure 6. Motility of free swimming cells and bacteriabots at two different viscosities.** **(A-D)** Distributions of swimming speed (A, B) and of chemotactic drift as a function of the swimming speed (C, D) were calculated from individual 2D trajectories for free swimming *E. coli* cells and cells attached to 2.2 µm particles (see Methods for details). Cells were measured in motility buffer with and without the addition of 0.25 % w/v 4000 cP methyl cellulose (~4.3 mPa·s at 32°C)[^71^](#_ENREF_71). In the viscous fluid, average chemotactic drift was 3.44 ± 0.22 µm/s (± SEM) for free swimming cells, and 0.12 ± 0.09 µm/s for bacteriabots. Numbers of analyzed trajectories in buffer without and with methyl cellulose were 109192, 3068 for free-swimming cells and 30724, 2924 for bacteriabots. Error bars show SEM.


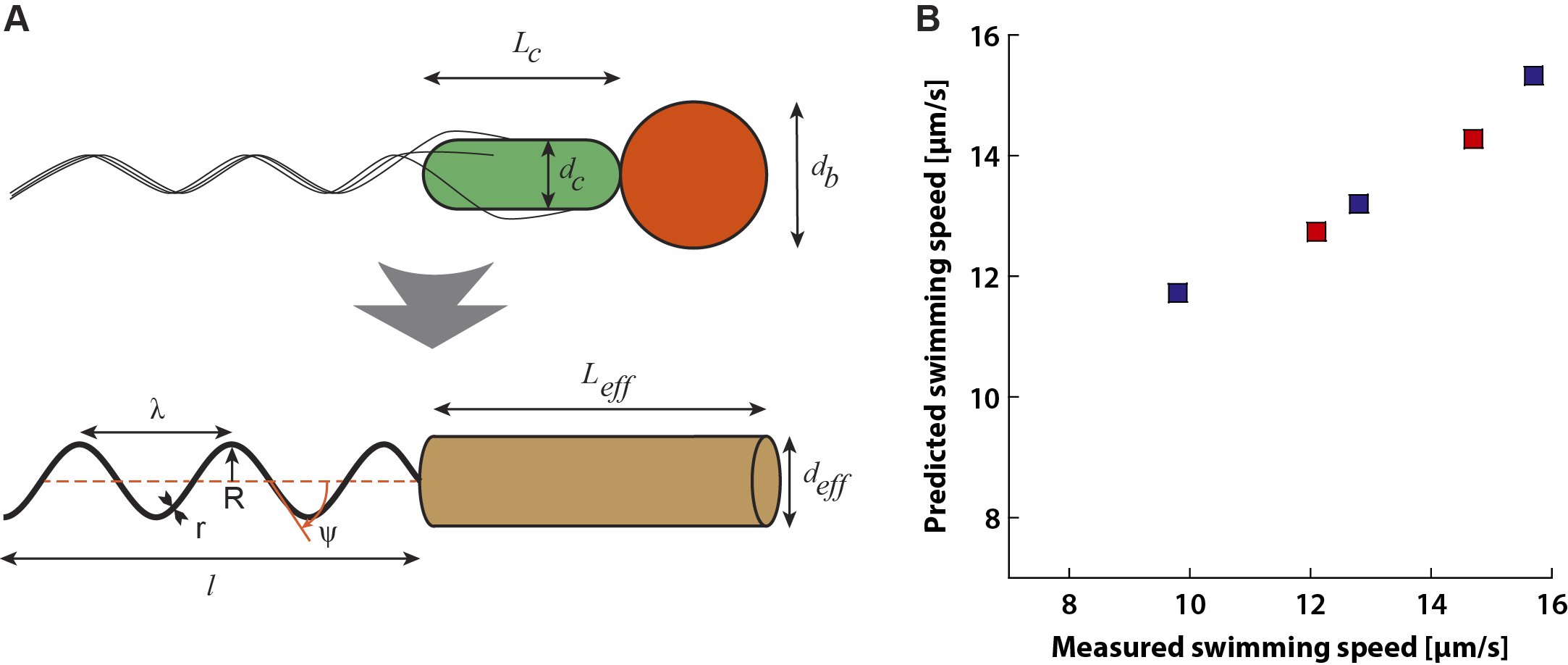


**Supplementary Figure 7. Analysis of cargo effects on swimming speed using resistive force theory. (A)** Schematic representation of a swimming bacteriabot used for theoretical analysis. The cell body of length *L_c_* and diameter *d_c_* and attached bead of diameter *d_b_* was modeled by an equivalent cylinder with the effective length *L_eff_* and diameter *d_eff_* (see Supporting Text for calculations). The flagellar bundle was modeled by a single helix, the thickness of which represents several single flagella. **(B)** Swimming speeds predicted by the resistive force theory (see Supporting Text) as a function of the measured swimming speed, compared to the experimental data for the five conditions studied (blue: normal cells, red: cephalexin-treated cells).


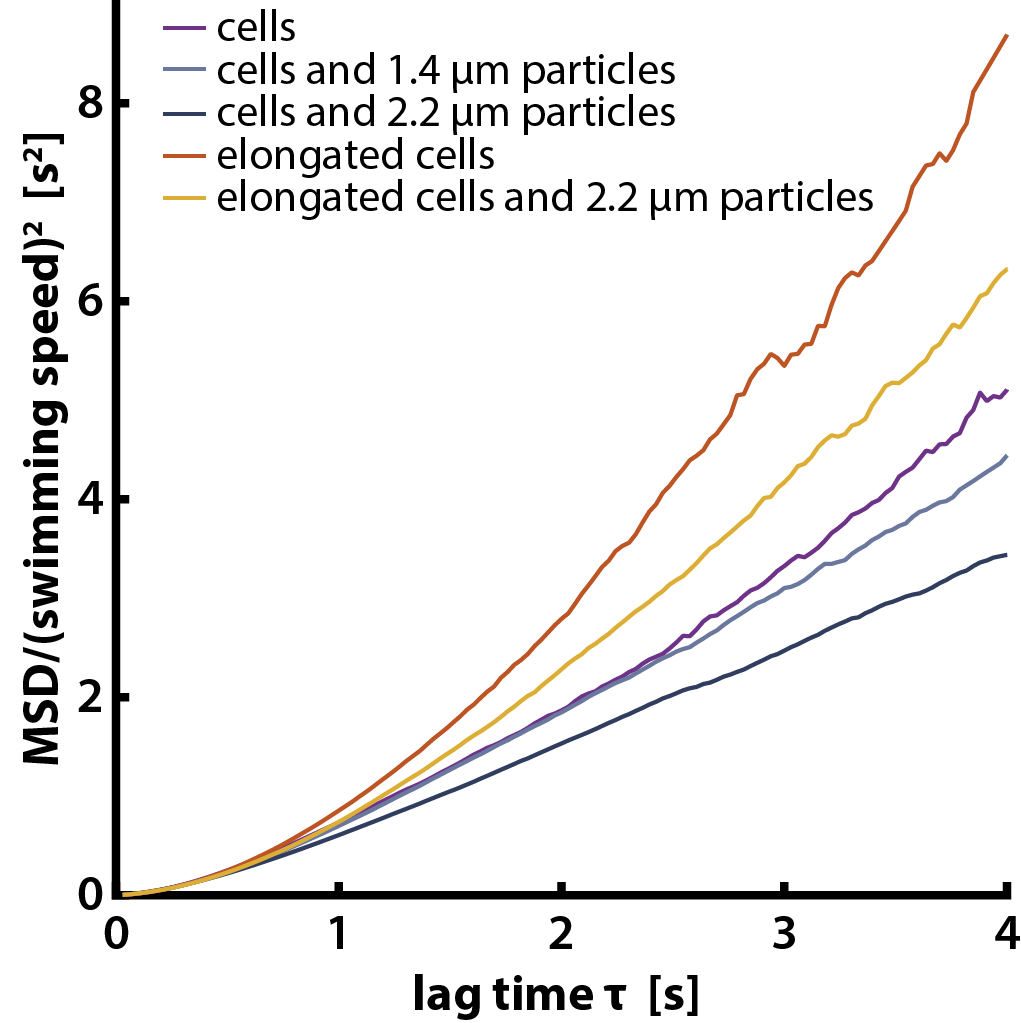


**Supplementary Figure 8. Mean-square displacement (MSD) of free cells and bacteriabots.** MSD normalized by the square of the swimming speed was calculated from the trajectory data in Fig. 3 as described in Methods.


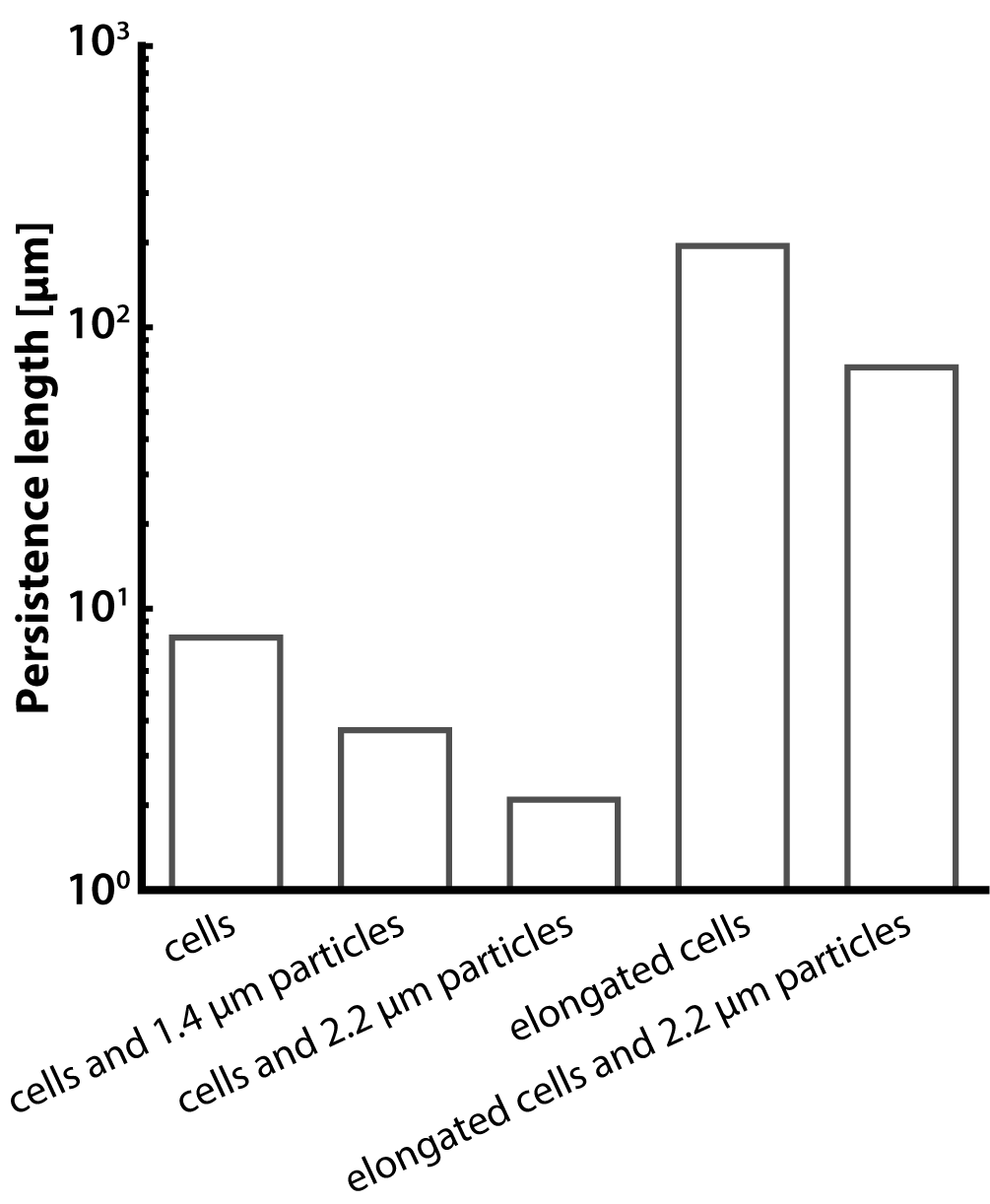


**Supplementary Figure 9. Persistence length of cells and bacteriabots.** The persistence length was obtained by fitting data in Fig. 3G as described in Methods.


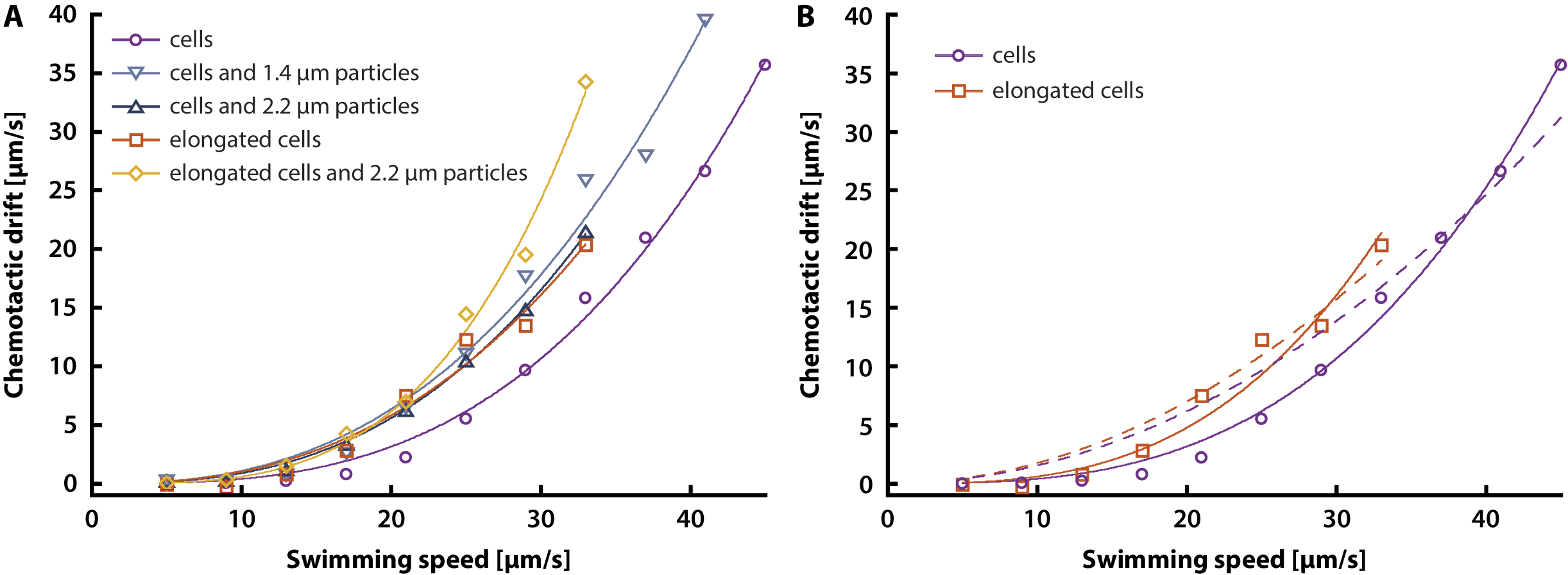


**Supplementary Figure 10. Fits of the chemotactic drift as a function of swimming speed. (A)** Fit by a power law $v_{ch} = K_{\alpha} v_{0}^{\alpha}$ yielded exponents close to 3: $\alpha$ = $3.0\pm0.1$ (normal cells), $2.5\pm0.2$ (normal cells and 1.4 µm particles), $2.7\pm0.1$ (normal cells and 2.2-µm particles), $2.5\pm0.3$ (elongated cells), $3.4\pm0.2$ (elongated cells and 2.2-µm particles), with $K_{\alpha}$ = $0.4$ (normal cells), $3$ (normal cells and 1.4-µm particles), $2$ (normal cells and 2.2-µm particles), $3$ (elongated cells), $0.2$ (elongated cells and 2.2-µm particles), in ${10}^{-3} \left( \text{μm}/\text{s} \right)^{1-\alpha}$. **(B)** Fits by a square ($v_{ch} = K_{2} v_{0}^{2}$, dotted line) and cubic ($v_{ch} = K_{3} v_{0}^{3}$, solid line) dependence of the chemotactic drift on swimming speed, for normal cells ($K_{2}=0.015\pm0.001$ s/µm, $R_{2}=0.970$, $K_{3}=\left( 3.95\pm0.07 \right){10}^{-4}$ s^2^/µm^2^, $R_{3}=0.997$) and elongated cells ($K_{2}=0.017\pm0.001$ s/µm, $R_{2}=0.977$, $K_{3}=\left( 6.0\pm0.3 \right){10}^{-4}$ s^2^/µm^2^, $R_{3}=0.981$).


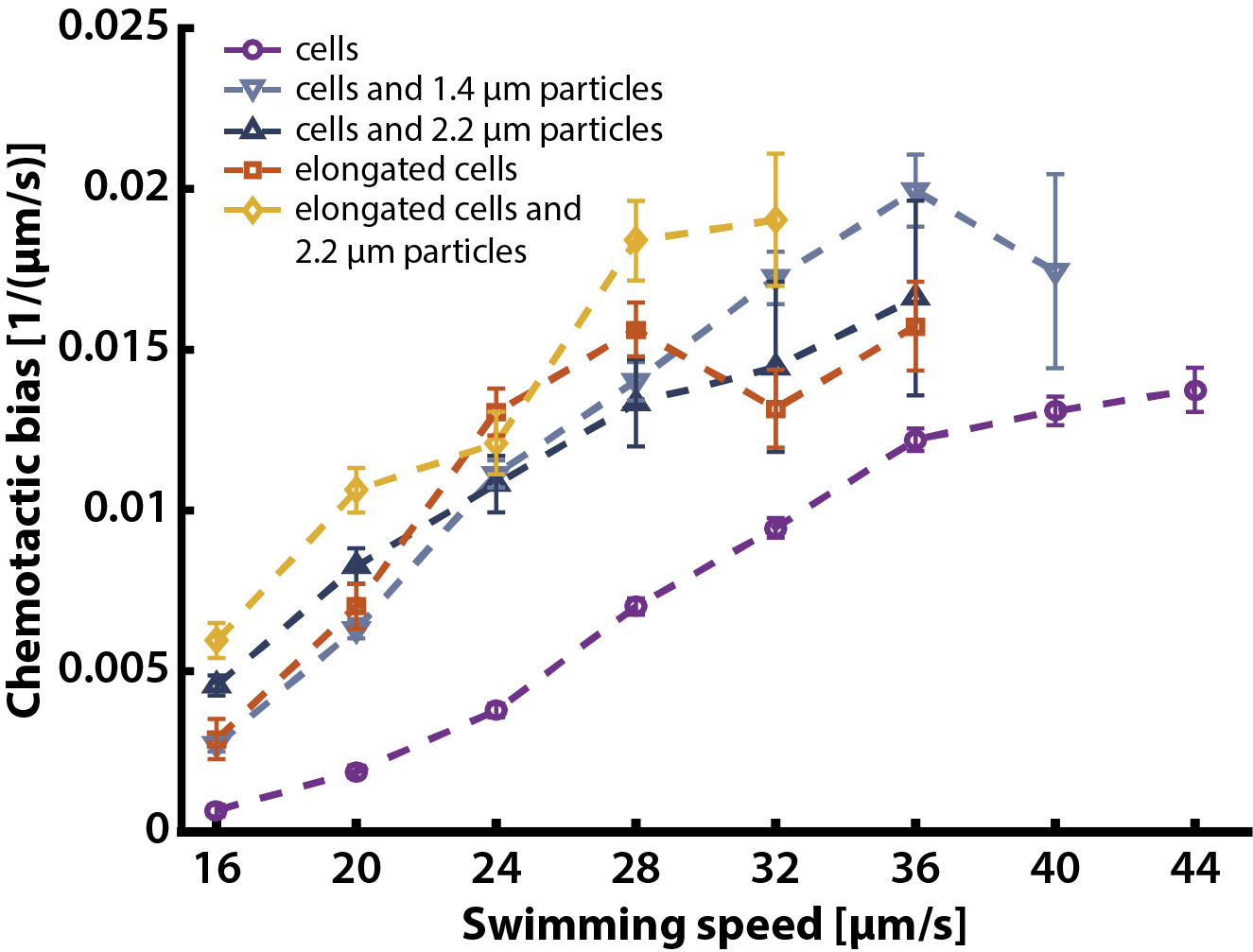


**Supplementary Figure 11. Chemotactic bias of free cells and bacteriabots as a function of the swimming speed.** Chemotactic bias, defined as chemotactic drift normalized by the swimming speed, $v_{ch}/v_{0}^{2}$, was calculated as described in Methods from the trajectory data in Fig. 4. Error bars show SEM.

Supplementary Table S1. Parameters used for calculations of swimming speed in the model

| **Parameter** | **Value** | **Reference** |
| --- | --- | --- |
| $\lambda$ | 2.3 µm | [^73^](#_ENREF_73) |
| $R$ | 0.2 µm | [^73^](#_ENREF_73) |
| $r$ | 40 nm | [^68^](#_ENREF_68) |
| $\gamma_{k}$ | 0.7 | [^50^](#_ENREF_50) |
| $\sin\psi$ | 0.88 | $\tan\psi=\lambda/{2\pi R}$ |
| $\omega^{m}$ | $2\pi\times140$ rad/s | [^49^](#_ENREF_49) |
| $l$, cpx- | 10 µm | [^68^](#_ENREF_68) |
| $l$, cpx+ | 17 µm | This study |

**
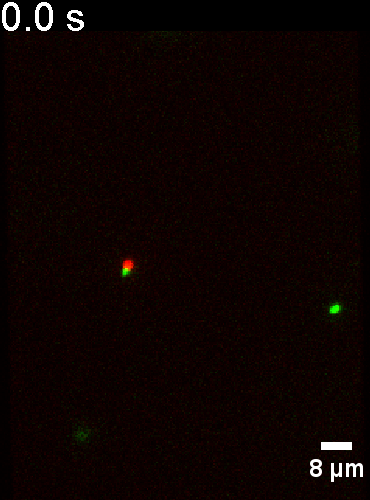
**

**Supplementary Movie 1. Bacteriabot motility: one bacterium attached to one particle.** For detailed informations see Fig. 2D.

**
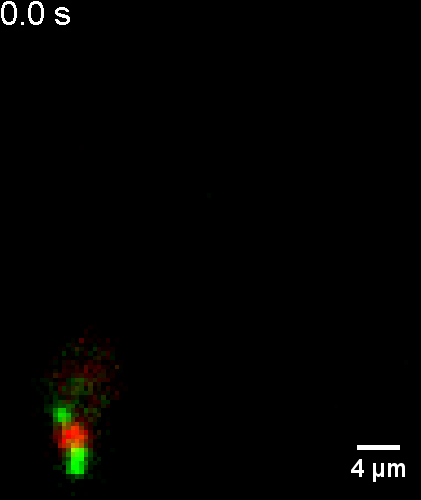
**

**Supplementary Movie 2. Bacteriabot motility: two bacteria attached to one particle (aligned).** For detailed informations see Fig. 2D.

**
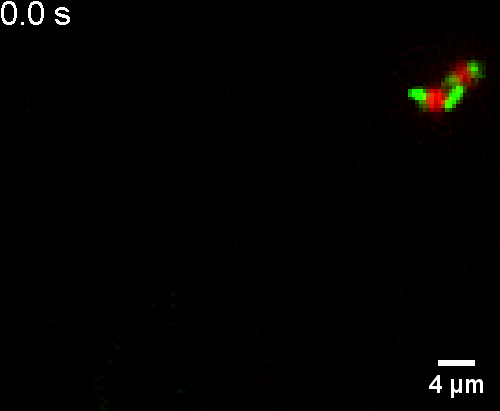
**

**Supplementary Movie 3. Bacteriabot motility: three bacteria attached to two particles (aligned).** For detailed informations see Fig. 2D.

**
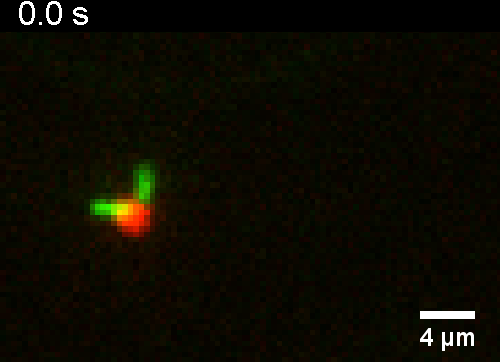
**

**Supplementary Movie 4. Bacteriabot motility: two bacteria attached to one particle (non-aligned).** For detailed informations see Fig. 2D.

**
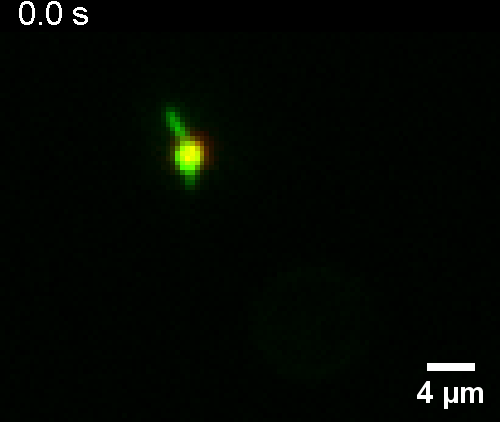
**

**Supplementary Movie 5. Bacteriabot motility: three bacteria attached to one particle (non-aligned).** For detailed informations see Fig. 2D.

**
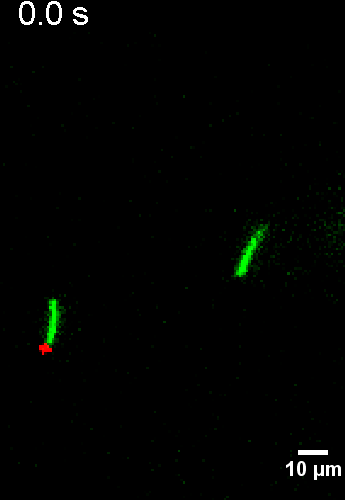
**

**Supplementary Movie 6. Bacteriabot motility: one elongated bacterium attached to one particle.** For detailed informations see Fig. 3F.

# References

48. Purcell, E. M. The efficiency of propulsion by a rotating flagellum. *Proc Natl Acad Sci USA* **94**, 11307-11311 (1997).

49. Guadayol, O., Thornton K. L. & Humphries S. Cell morphology governs directional control in swimming bacteria. *Sci Rep* **7**, 2061 (2017).

50. Chattopadhyay, S., Moldovan R., Yeung C. & Wu X. L. Swimming efficiency of bacterium *Escherichia coli*. *Proc Natl Acad Sci USA* **103**, 13712-13717 (2006).

68. Purcell, E. M. Life at Low Reynolds-Number. *Am J Phys* **45**, 3-11 (1977).

69. Tirado, M. M., Martinez C. L. & Delatorre J. G. Comparison of Theories for the Translational and Rotational Diffusion-Coefficients of Rod-Like Macromolecules - Application to Short DNA Fragments. *J Chem Phys* **81**, 2047-2052 (1984).

70. Chen, X. & Berg H. C. Torque-speed relationship of the flagellar rotary motor of *Escherichia coli*. *Biophys J* **78**, 1036-1041 (2000).

71. Berg, H. C. & Turner L. Movement of microorganisms in viscous environments. *Nature* **278**, 349-351 (1979).

72. Espinosa-Garcia, J., Lauga E. & Zenit R. Fluid elasticity increases the locomotion of flexible swimmers. *Phys Fluids* **25**, 031701; 10.1063/1.4795166 (2013).

73. Darnton, N. C., Turner L., Rojevsky S. & Berg H. C. On torque and tumbling in swimming *Escherichia coli*. *J Bacteriol* **189**, 1756-1764 (2007).
